# Modeling the Intra- and Extracellular Cytokine Signaling Pathway under Heat Stroke in the Liver

**DOI:** 10.1371/journal.pone.0073393

**Published:** 2013-09-05

**Authors:** Maria Rodriguez-Fernandez, Benyamin Grosman, Theresa M. Yuraszeck, Bryan G. Helwig, Lisa R. Leon, Francis J. Doyle III

**Affiliations:** 1 Institute for Collaborative Biotechnologies, University of California Santa Barbara, Santa Barbara, California, United States of America; 2 Department of Chemical Engineering, University of California Santa Barbara, Santa Barbara, United States of America; 3 Thermal and Mountain Medicine Division, United States Army Research Institute of Environmental Medicine, Natick, Massachusetts, United States of America; Indiana University School of Medicine, United States of America

## Abstract

Heat stroke (HS) is a life-threatening illness induced by prolonged exposure to a hot environment that causes central nervous system abnormalities and severe hyperthermia. Current data suggest that the pathophysiological responses to heat stroke may not only be due to the immediate effects of heat exposure *per se* but also the result of a systemic inflammatory response syndrome (SIRS). The observation that pro- (*e.g.*, IL-1) and anti-inflammatory (*e.g.*, IL-10) cytokines are elevated concomitantly during recovery suggests a complex network of interactions involved in the manifestation of heat-induced SIRS. In this study, we measured a set of circulating cytokine/soluble cytokine receptor proteins and liver cytokine and receptor mRNA accumulation in wild-type and tumor necrosis factor (TNF) receptor knockout mice to assess the effect of neutralization of TNF signaling on the SIRS following HS. Using a systems approach, we developed a computational model describing dynamic changes (intra- and extracellular events) in the cytokine signaling pathways in response to HS that was fitted to novel genomic (liver mRNA accumulation) and proteomic (circulating cytokines and receptors) data using global optimization. The model allows integration of relevant biological knowledge and formulation of new hypotheses regarding the molecular mechanisms behind the complex etiology of HS that may serve as future therapeutic targets. Moreover, using our unique modeling framework, we explored cytokine signaling pathways with three *in silico* experiments (*e.g.* by simulating different heat insult scenarios and responses in cytokine knockout strains *in silico*).

## Introduction

Heat stroke (HS) is a life-threatening illness characterized by profound central nervous system dysfunction, severely elevated core temperature, as well as organ and tissue damage resulting from environmental heat exposure [1]. Environmental heat exposure is one of the most deadly natural hazards in the United States with ∼200 deaths per year. In the past two decades, extreme heat exposure claimed more American lives than the combined effects of hurricanes, lightning, earthquakes, floods and tornadoes [2]. HS is also an international hazard as demonstrated by the high incidence of death (>15,000 individuals) during the 2003 heat wave in France [3], [4]. Clinical and experimental evidence suggests that the pathophysiological responses to HS are the result of a systemic inflammatory response syndrome (SIRS) that ensues following HS collapse. The SIRS is regarded as a response to bacteria and/or endotoxin leakage across ischemic-damaged gut epithelial barrier membranes, which stimulates cytokine and other inflammatory pathways that are thought to mediate a variety of pathophysiological responses. The liver has been implicated as an early key player in the heat-induced SIRS based on its function as a major site of endotoxin clearance [5]. Cytokines are important regulators of the acute-phase response (APR) to inflammation/injury and have been implicated as mediators of the SIRS with HS [6].

Several studies have characterized peripheral cytokine disturbances in HS patients. At the time of clinical admission or shortly after cooling, the concentration of circulating interleukin (IL)-1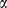, IL-1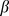, IL-1 receptor antagonist (IL-1Ra), IL-6, soluble IL-6 receptor (sIL-6R), IL-10, interferon (IFN)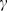, tumor necrosis factor (TNF)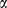, and/or soluble TNF receptors subtype I (sTNF-RI) and subtype II (sTNF-RII) have been shown to be elevated in some HS patients [7]–[10]. Unfortunately, circulating cytokines are often determined primarily at end-stage HS, which has limited our understanding of the time course of changes in the balance of these mediators during progression of the SIRS. Moreover, the complex interactions among cytokines that mediate the APR and SIRS remain unknown. Development of a conscious mouse model that simulates the human pathophysiological responses to HS has demonstrated that plasma concentrations of IL-1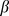, IL-6, IL-10, and IL-12p40 are increased in a time- and core temperature (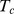)-dependent manner [6]. Furthermore, concomitant elevation of pro- (e.g., IL-1) and anti-inflammatory (e.g., IL-10) cytokines suggests that a complex network of interactions orchestrates the SIRS during HS recovery.

Accompanying elevations in cytokines, organ (kidney, liver, spleen) and tissue (gut, skeletal muscle) damage are common manifestations of the HS syndrome [11]–[13]. The liver is a major immune organ known to produce and respond to cytokines during inflammation [14] and damage to this organ is primarily observed in long-term survivors of HS [15]. However, it is unknown if liver damage is a consequence of direct thermal injury or cytokine-induced pathophysiological changes associated with the SIRS, indicating the importance of correlating changes in circulating cytokine levels with inflammatory changes occurring at the organ and/or tissue level. Helwig and Leon [14] determined plasma, liver, and spleen mRNA accumulation patterns for the IL-1 family members in mice following HS; increased IL-1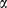, IL-1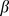, and IL-1 receptor subtype I (IL-1RI) and subtype II (IL-1RII) mRNA accumulation in the liver and spleen suggested these organs may contribute to circulating IL-1 family protein levels following HS, but the absence of studies on protein translation that include protein tagging precluded a conclusive association.

TNF-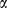 has been shown to have deleterious actions in endotoxemia [16], [17] and it has been assumed that this cytokine functions similarly in HS. Leon [18] conducted the first studies using TNF receptor subtype I (TNF-RI) and subtype II (TNF-RII) knockout mice (TNFR KO; *i.e.*, can produce TNF, but do not have signaling receptors) and showed slower heating and faster cooling rates in KO compared to wild-type mice. Although the 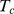 responses displayed by TNFR KO mice would be considered protective against HS, these mice showed a trend towards increased mortality compared to their wild-type controls during the second day of recovery (40% vs. 100% survival, respectively). This preliminary study indicates that TNF might have time-dependent pro- (early) and anti-inflammatory (late) actions in the HS syndrome, although the mechanisms mediating the early actions of this cytokine in HS remain unidentified. It is important to elucidate the pro-inflammatory actions of TNF in the heat-induced SIRS to determine if this cytokine may be an important therapeutic target to mitigate morbidity/mortality associated with this syndrome.

Using a conscious mouse HS model, we showed previously that several pro- and anti-inflammatory cytokines are elevated in the circulation and liver following HS collapse [14], [18]. TNF is known to interact with several other cytokine pathways during bacterial infection and it is assumed that similar mechanisms of inflammation are mediating the SIRS to HS. Therefore, using a conscious mouse HS model we measured circulating cytokine/soluble cytokine receptor (IL-1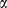, IL-1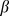, IL-6, IL-10, TNF-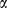, sIL-1RI, sIL-1RII, sIL-6R, sTNF-RI, and sTNF-RII), liver cytokine and receptor mRNA accumulation (IL-1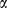, IL-1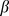, IL-6, IL-10, TNF-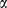, IL-1RI, IL-1RII, IL-6R, TNF-RI, and TNF-RII), and liver HSP70 mRNA accumulation in wild-type (WT) and TNFR KO mice. HSP70 mRNA accumulation levels were used as a sensitive measure of stress to this organ, as this pathway is activated by many factors that are inherent in our HS model (e.g., heat stress, dehydration, oxidative stress).

The mediators involved in progression of the HS syndrome and the intricate map of interactions between them form a complex system that can only be truly understood using a systems approach. Although several computational models of acute inflammation exist [19], [20], the aim of this study was to incorporate a level of mechanistic detail that was not previously incorporated into these models. Therefore, we developed a mathematical model that integrates relevant biological knowledge with our novel experimental data from wild-type mice to identify testable hypotheses that will delineate the molecular mechanisms mediating the complex etiology of the heat-induced SIRS. This mechanistic dynamic model describes intra- and extracellular changes (in the liver and in the plasma, respectively) in cytokine signaling pathways under HS and was fitted to genomic and proteomic data of wild-type mice by means of global optimization techniques. Model validation was performed using a completely different set of data from TNFR KO mice that were not used for calibration purposes, but demonstrate the predictive capabilities of our framework. The broader applicability of the developed model in the context of acute inflammation was assessed by comparing its predictions with experimental data from mice treated with LPS [21]. The purpose of this study was to: 1) assess the complex interaction of cytokines in the liver and the circulation during early progression of the heat-induced SIRS; 2) gain insight into molecular mechanism(s) that may serve as future therapeutic targets for HS patients; 3) analyze the correlation of organ (liver) mRNA accumulation and circulating levels of cytokines; 4) determine whether the TNFR KO responses in the liver and the circulation are altered due to differences in heating and cooling or represent a direct effect of the absence of TNF signaling. Moreover, we provide a unique modeling framework that supports identification of the role of different cytokine signaling pathways using three *in silico* experiments that can be used to guide further *in vivo* experiments. Specifically, we examined the response to the exposure to a more severe heat insult, the injection of a dose of LPS, and the knockout of IL-10R.

## Results

### Mathematical Model of the Cytokines Network

The primary mechanisms represented in this model are the following: activation of various transcription factors (TFs) by stimulation via a set of external and internal signals triggered by heat stress; regulation of cytokine and cytokine receptor gene transcription involved in the network by means of these TFs; translation of mRNA into proteins; transport of the soluble proteins to the pericellular milieu; binding of plasma cytokines to their cognate receptors; and signaling back to the TFs. Details about the modeling assumptions are given in the *Methods* section.

**Figure 1:**
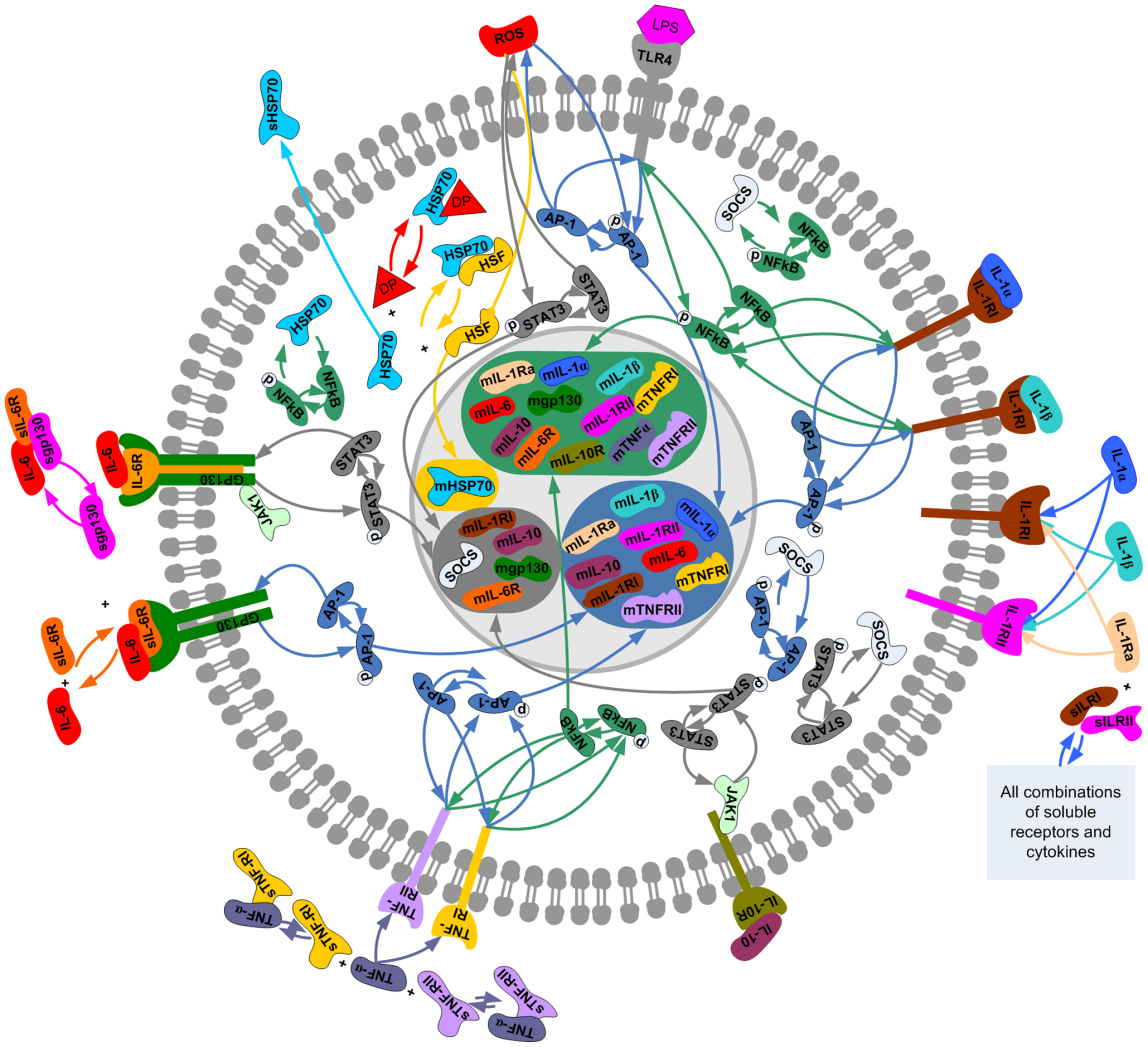
Cellular network of interactions amongst HSP70, TLR4, IL-1, IL-6, IL-10, and TNF families induced by heat stroke. Prolonged exposure to a high ambient temperature increases core temperature and is associated with organ damage, increase of denatured proteins (DP), reactive oxygen species (ROS), and LPS. As a consequence, transcription factors (TF) HSF-1, NF-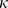
B, AP-1, and STAT-3 are activated and regulate the transcription of a set of cytokine genes in the cell nucleus represented by the inner grey circle (each of the colored boxes contains the genes regulated by a certain TF: HSF in yellow, NF-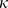
B in green, AP-1 in blue, and STAT-3 in dark grey). These mRNAs are exported to the cytoplasm and translated into proteins (not represented in the figure). These cytokines/soluble cytokine receptors can exit the cell throughout the cell membrane (outer grey circle) and be released into the blood stream. The proteins that are embedded into the cell membrane represent transmembrane receptors.

The elevated 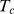 in response to heat insult is one of several factors that is thought to induce organ damage and increase the concentration of denatured proteins (DP), endotoxins (lipopolysaccharide, LPS), and reactive oxygen species (ROS) that concomitantly initiate a network of cytokine responses. Four TFs were assumed to be the primary regulators of this network, namely, heat shock factor 1 (HSF-1), nuclear factor-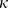
B (NF-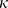
B), activator protein 1 (AP-1), and signal transducer and activator of transcription 3 (STAT-3). The implicated ligands and receptors were classified into six families (heat shock protein (HSP)70, toll-like receptor (TLR)-4, IL-1, IL-6, IL-10, and TNF) and their inter- and intracellular activity was incorporated into the model as six interconnected modules (Figure 1 and Supporting Information S2).

The model is described by a system of 65 ordinary differential equations (ODEs) and 217 parameters from which 130 parameters were fitted to experimental data corresponding to a strain of WT mice (B6129F_2_) using the toolbox SensSB [22]. The model was built based on the hypothesis that the liver is the main source of circulating cytokines. In order to test this hypothesis, during model fitting we gave priority to the liver qPCR data (higher weights in the cost function); therefore, we tried to achieve the best possible fitting for the liver and analyze whether it was possible to simultaneously fit the circulating cytokines based on the aforementioned hypothesis. For the mRNA expression of the WT animals, the fitting lies within the confidence interval of almost every experimental point. Only the values of mIL6 and mIL10 for 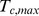 are considerably smaller than those predicted by the model; disregarding these two points, the average of the absolute values of the standardized residuals (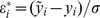) is smaller than one, meaning that, on average, the error of the fit is smaller than one standard deviation from the mean [23]. The model was validated using a set of data corresponding to TNFR KO mice that was not used for model calibration. Note that, for validation purposes, all parameters were fixed to the best values obtained in the calibration step except for those related to TNF-RI and II transcription, which were set to zero. A file with the value of the parameters, reaction rates, and model equations is included as Supporting Information S3. SensSB files needed to reproduce the results are provided as Supporting Information S4. Matlab figures containing the estimated time course for all the states are available as Supporting Information S5.

### Experimental Data, Model Fitting and Validation

Liver mRNA accumulation and circulating cytokines and receptors concentration were measured at four sampling points: 1) baseline (prior to heat exposure); 2) maximum core temperature (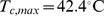), also referred to as HS collapse, at which time the mice were removed from the heat; 3) ∼30 minutes of HS recovery when the core temperature returns to baseline (RTB; first 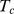 value 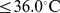 during cooling); 4) ∼3 hours of recovery when mice exhibit hypothermia depth (lowest 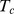 value with cooling rate of 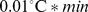 during recovery). Liver mRNA accumulation of AP-1 and NF-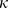
B related genes is shown in Table 1. Liver mRNA accumulation of HSP, cytokines, and cytokine receptors is summarized in Table 2. The experimental protocol is detailed in the *Methods* section.

**Table 1:** Liver fold-change in mRNA accumulation during heat stroke recovery in WT and TNFR KO mice for NF-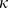
B and AP-1 related genes.

Gene	Strain	T*_C,max_*	RTB	Hypothermia
NF- 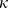 B1	B6129F2	**0.750**	0.872	1.14† ^a^
	TNFR KO	0.846	1.04	1.29† § ^a^
				
NF- 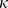 B2	B6129F2	**1.37** a	1.65	**1.86**
	TNFR KO	**1.55** a	**2.08**	**2.06** †
				
RELA	B6129F2	1.08	1.08	**1.46** †
	TNFR KO	1.18	1.37	1.38
				
RELB	B6129F2	1.55	**2.02**	**2.03**
	TNFR KO	**2.14** §	2.14	**3.16** †
				
C-REL	B6129F2	**4.26**	**3.75**	**4.27**
	TNFR KO	**4.54**	**3.69**	**4.32**
				
I 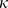 B	B6129F2	1.30	1.36	**1.67**
	TNFR KO	1.05	1.42	1.52
				
JUN	B6129F2	**53.0** a	**109.9** †	**41.7**
	TNFR KO	**40.8** a	**86.9** †	**93.0** †
				
FOS	B6129F2	**46.7**	**52.8**	**24.2** a
	TNFR KO	**53.6**	**53.6**	**115.9** † ^a^

Data represent fold-change in liver mRNA accumulation relative to controls at the same time point. Bold fonts represent significantly higher than time-matched controls (one-way ANOVA, P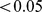);

† represents significant difference from T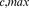;

§ represents significant difference from return-to-baseline;

a represents significant difference between genotypes within each gene. T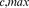, maximum core temperature (42.4°C); TNFR KO, tumor necrosis factor receptor knockout; WT, wild-type strain (B6129F_2_).

**Table 2:** Liver fold-change in mRNA accumulation during heat stroke recovery in WT and TNFR KO mice for HSP, cytokines, and cytokine receptors.

Gene	Strain	T*_C,max_*	RTB	Hypothermia
HSP70	B6129F2	**657.9**	**780.4** †	**1204.5** † §
	TNFR KO	**565.8**	**783.2** †	**1071.1** † §
				
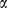 IL-1	B6129F2	**2.0** a	**3.0** a	**2.1**
	TNFR KO	1.4§ ^a^	**1.6** † ^a^	**3.7** †
				
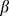 IL-1	B6129F2	**2.6** a	**10.7** † ^a^	**4.7**
	TNFR KO	**2.2** a	**4.7** † ^a^	**7.0** †
				
IL-1RI	B6129F2	**3.1**	**3.0**	**9.0** † §
	TNFR KO	**3.0**	**2.9**	**4.7** †
				
IL-1RII	B6129F2	0.6^a^	**1.9** a	**9.4** † §
	TNFR KO	0.5^a^	0.8† ^a^	**6.3** † §
				
IL-6	B6129F2	1.1§ ^a^	**14.9** † ^a^	**2.7** § ^a^
	TNFR KO	0.9§ ^a^	**6.7** † ^a^	**6.4** † ^a^
				
IL-6R	B6129F2	1.2	1.1	**1.7** §
	TNFR KO	1.2	1.2	**1.4** †
				
gp130	B6129F2	**1.4**	1.0	**1.5**
	TNFR KO	1.2	1.4	1.2
				
IL-10	B6129F2	1.0	**11.3** † ^a^	**14.0** †
	TNFR KO	1.1	**4.6** † ^a^	**19.3** † §
				
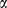 TNF-	B6129F2	**0.4**	2.0†	1.1†
	TNFR KO	0.5	0.7	0.8

Data represent fold-change in liver mRNA accumulation relative to controls at the same time point. Bold fonts represent significantly higher than time-matched controls (one-way ANOVA, P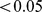);

† represents significant difference from T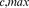;

§ represents significant difference from return-to-baseline;

a represents significant difference between genotypes within each gene. T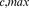, maximum core temperature (42.4°C); TNFR KO, tumor necrosis factor receptor knockout; WT, wild-type strain (B6129F_2_).

#### Mouse core temperature profiles

**Figure 2:**
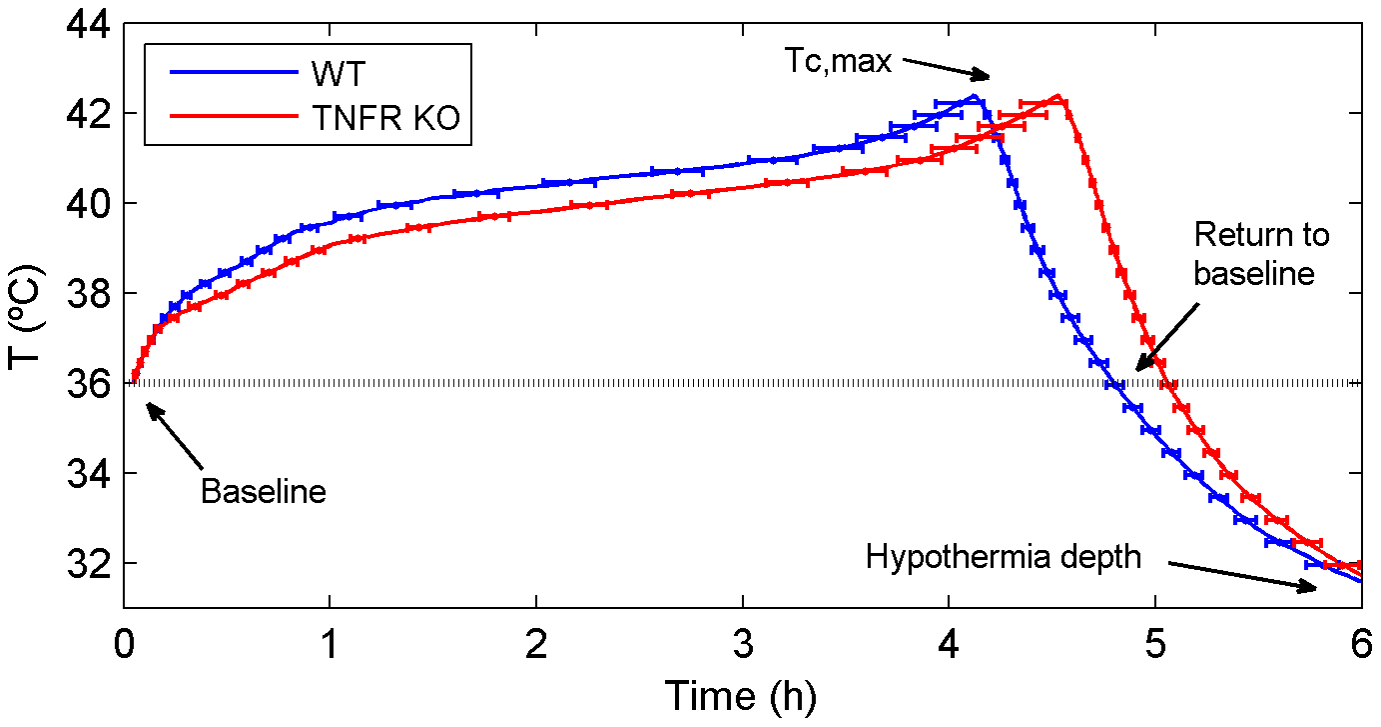
Averaged core temperature responses for WT and TNFR KO mice during heat exposure and recovery at a constant ambient temperature. Experimental data were collected at four different points based on mouse core temperature responses (baseline 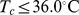, 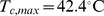, return to baseline, and hypothermia depth); therefore, the average was computed along the temperature axis.

The two strains of mice used in this study, WT and TNFR KO, showed significantly different 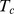 profiles under the same heat stress protocol (Figure 2). TNFR KO mice maintained ∼0.6°C lower 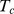 than WT mice during hyperthermia but experienced similar thermal load (a measure of heat strain), although the time to reach 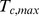 was ∼30 minutes longer than the WT mice (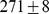
% vs. 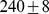
%, respectively; ANOVA, 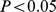) and associated with a significantly higher level of dehydration (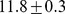
% vs. 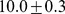
%, respectively; ANOVA, 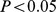) [24]. TNFR KO mice also showed a significantly faster cooling rate than WT mice from 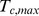 to hypothermia depth. Despite genotype differences in heating and cooling profiles, hypothermia depth was virtually identical between genotypes and was observed ∼3 hours after removal from the heat stress environment. Taken together, these data indicate a direct effect of TNF signaling on 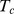 regulation during heat exposure and recovery [24].

#### Liver transcription factor mRNA accumulation

We measured liver mRNA accumulation of all the NF-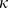
B family members, namely, NF-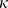
B1, NF-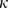
B2, RELA, RELB, C-REL, and I
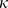
B (see Table 1). Liver NF-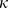
B1, NF-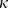
B2, RELA, and I
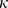
B mRNA accumulation was not significantly or little altered (less than two-fold) at any time point in either genotype. C-REL was upregulated ∼4-fold at all sampling time points after the onset of HS (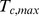, RTB, and hypothermia depth) and RELB was ∼2-fold increased at RTB and hypothermia with less mRNA accumulation observed at 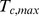 for the WT mice. Although we did not directly measure NF-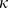
B nuclear localization, some studies suggest that increased RELB transcription and translation is a direct measure of this activity [25]. Overall, we did not detect any genotype differences in liver mRNA accumulation of the NF-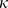
B family members.

The most robust changes between genotypes in the liver were observed in JUN and FOS mRNA accumulation profiles (Table 1). While both TFs were significantly upregulated (fold-change ∼55.3 and ∼38.5, respectively) at 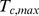, JUN mRNA accumulation was significantly lower at 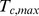 in TNFR KO compared to WT mice. Liver JUN mRNA accumulation reached peak levels at RTB in the WT mice (fold-change ∼123) whereas TNFR KO mice showed delayed mRNA accumulation with maximum values observed at hypothermia (fold-change ∼92). Interestingly, JUN and FOS mRNA accumulation at baseline was slightly but significantly lower (fold-change ∼0.8) in the liver of TNFR KO compared to WT mice suggesting homeostatic regulation of this response by TNF.

#### HSPs

Baseline liver HSP70 mRNA accumulation did not differ between genotypes and values observed in non-heated controls were similar across all time points (data not shown). At 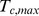, both genotypes showed ∼600-fold increase in liver HSP70 mRNA accumulation that was significantly higher than controls, similar between genotypes, and sustained through RTB in both groups (Table 2; one-way ANOVA, P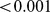). Peak mRNA accumulation of HSP70 was observed at hypothermia with no significant difference between genotypes (WT: 1364-fold; TNFRKO: 1034-fold; Table 2).

**Figure 3:**
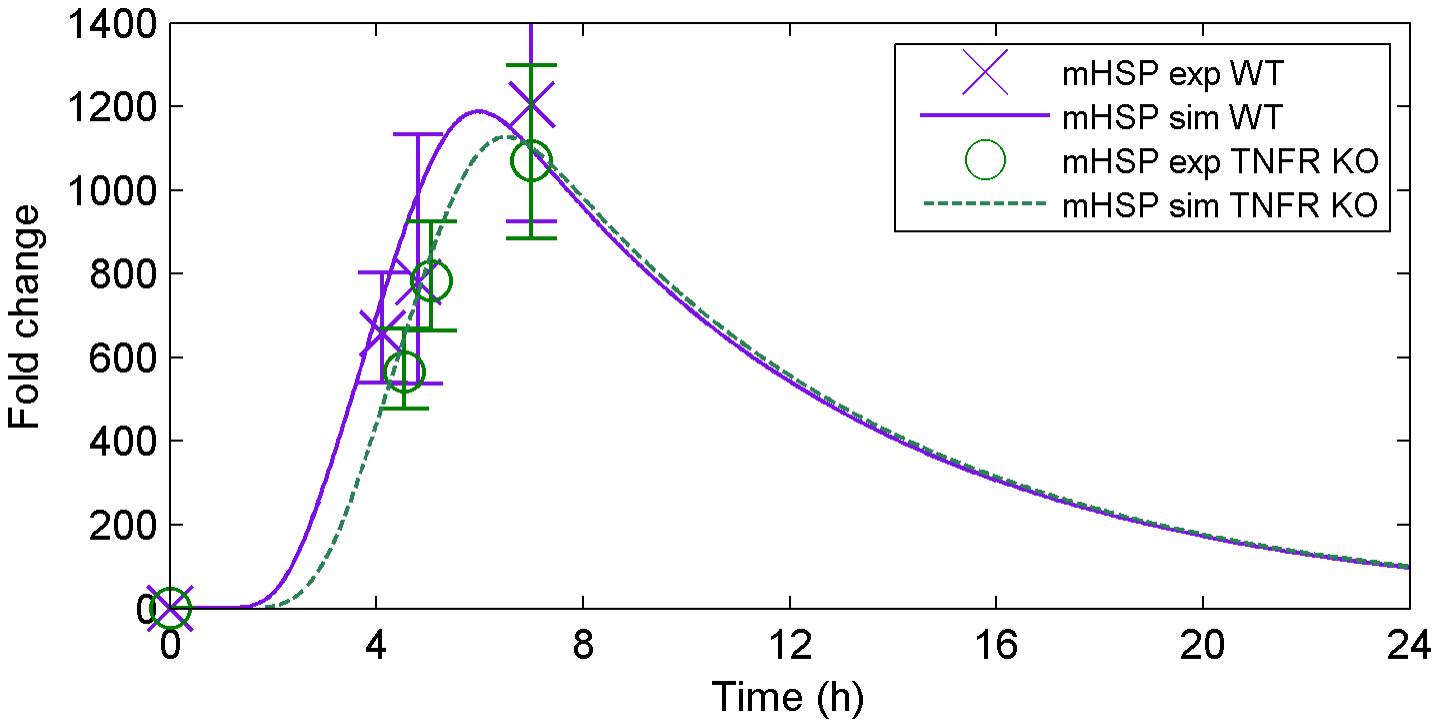
Model simulation (solid and dashed lines) versus experimental data (markers) for the mRNA accumulation of HSP70 under heat stroke. The model accurately fits the WT data; moreover, it predicts a slightly delayed and attenuated response for the TNFR KO mice in agreement with the experimental data (data used for validation only).

Figure 3 shows model prediction based on WT data versus experimental data from TNFR KO mice for liver mRNA accumulation of HSP70. In accordance with literature [26] as well as our experimental data, the model accurately predicts increased liver HSP70 mRNA accumulation shortly after HS collapse (∼4–4.5 hours), with peak mRNA accumulation (>1000 fold) occurring at ∼7 hours, when mice are hypothermic. As illustrated in Figure 3, our model accurately fits the WT data used for calibration (solid purple line) and, more importantly, predicts a slightly delayed and attenuated response of the TNFR KO strain (dashed green line). Although our experimental data from TNFR KO mice suggested attenuated liver HSP70 mRNA accumulation, this trend was not statistically significant. However, one might expect such a response to occur in the TNFR KO mice since the higher level of dehydration experienced by these mice would generate greater ROS production and consequent inhibition of HSF-1.

#### IL-1 family

**Figure 4:**
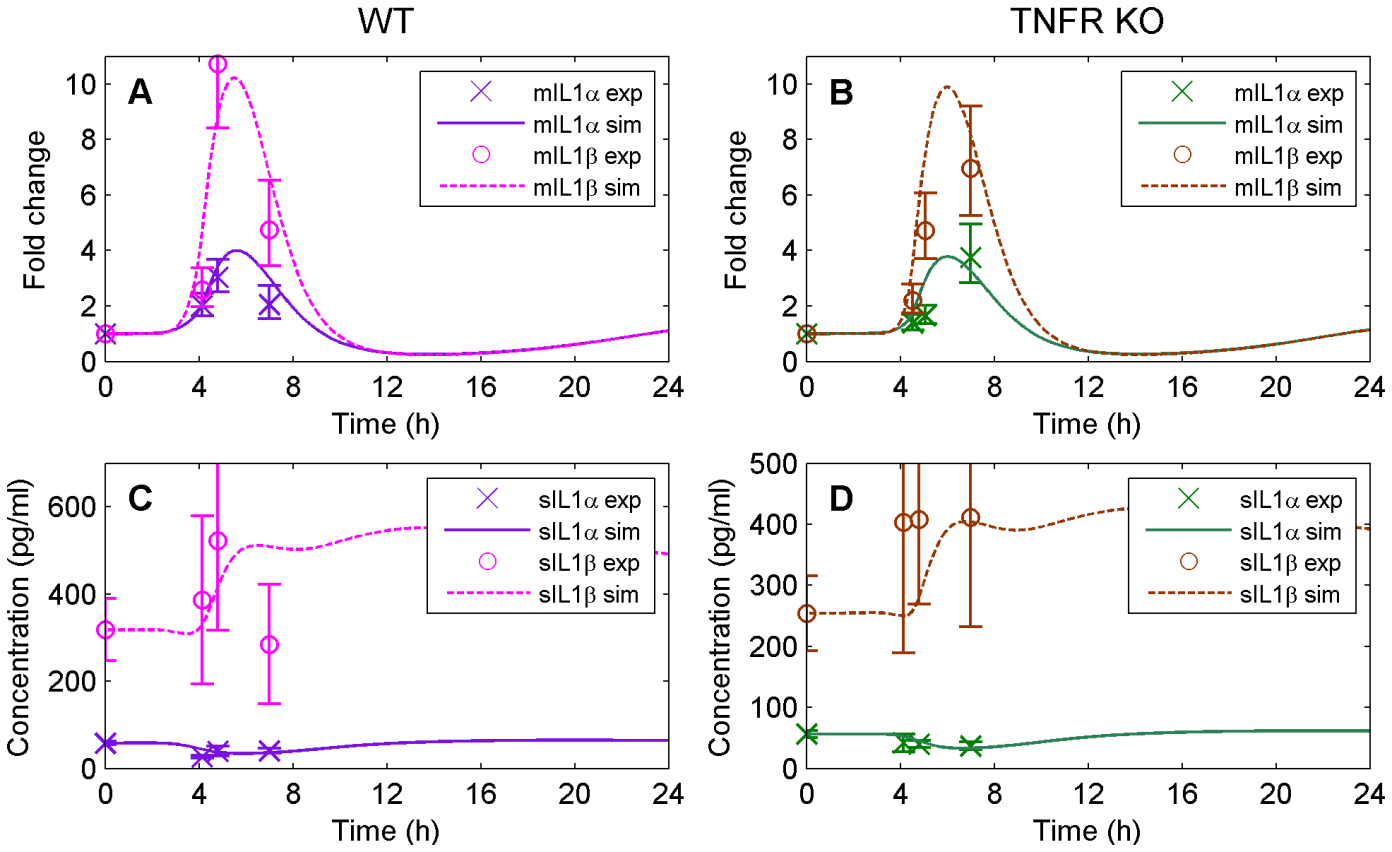
Model simulation (solid and dashed lines) versus experimental data (markers) for the IL-1 family. (A) represents the mRNA accumulation of IL-1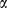 and IL-1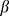 for WT, (B) the mRNA accumulation of IL-1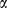 and IL-1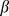 for TNFR KO (data used for validation only), (C) the plasma concentration of IL-1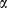 and IL-1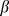 for WT, and, (D) the plasma concentration of IL-1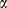 and IL-1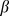 for TNFR KO (data used for validation only).

Figure 4 represents model predictions (solid and dashed lines) and experimental data (markers) for IL-1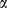 and IL-1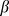 at the four sampling points (baseline, T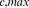, RTB, and hypothermia). The left panels (4A and 4C) correspond to model fitting using WT data, while right panels (4B and 4D) illustrate model validation using TNFR KO data that were not used for calibration. The top panels (4A and 4B) represent the fold change for liver mRNA accumulation (mIL-1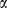, mIL-1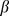) and the bottom panels (4C and 4D) correspond to the concentration of soluble (*i.e.*, circulating) cytokines (sIL-1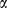, sIL-1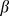).

IL-1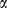, IL-1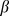, and IL-1Ra promoters contain NF-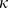
B and AP-1 regulatory elements [27], [28]. However, IL-1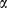 and IL-1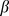 exhibited differential mRNA accumulation in the liver of HS mice, with IL-1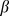 fold change significantly higher than IL-1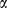 at all time points (see Table 2 and Figures 4A–B). Notably, liver IL-1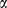 and IL-1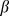 mRNA accumulation was significantly delayed in TNFR KO mice; these experimental data were captured by the model (Figure 4B). That is, our model predicted an earlier peak of mIL-1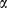 and 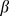 mRNA accumulation in the WT at RTB (third sampling point) at ∼5 hours while TNFR KO mice presented maximal mRNA accumulation between RTB and hypothermia (third and fourth sampling point) at ∼6 hours. IL-1RI mRNA accumulation was similarly elevated in both genotypes throughout recovery (∼3-fold increase), but showed peak mRNA accumulation at hypothermia (∼8-fold for WT and ∼4-fold for TNFR KO) compared to the earlier time points. IL-1RII mRNA accumulation was similar between groups with a significant increase in heated animals compared to controls at hypothermia only (fold change ∼6).

The concentration of sIL-1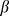 (*i.e.*, circulating protein) was ∼10-fold higher than sIL-1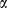 at all time points regardless of genotypes (Figures 4C–D). This difference in plasma levels might be due to the different signaling mechanisms of IL-1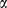 and IL-1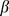 that induce expression of their own genes in an autocrine and paracrine manner, respectively [29]. In addition, HS had no significant effect on sIL-1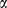 sIL-1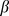, sIL-1RI or sIL-1RII levels in either genotype. The dissociation between liver IL-1 family mRNA accumulation and circulating protein levels, reproduced by the computational model, suggests that the proteins generated in the liver are not extensively released into the blood stream or present a long delay not captured within the time frame of our experimental protocol.

#### IL-6 family

**Figure 5:**
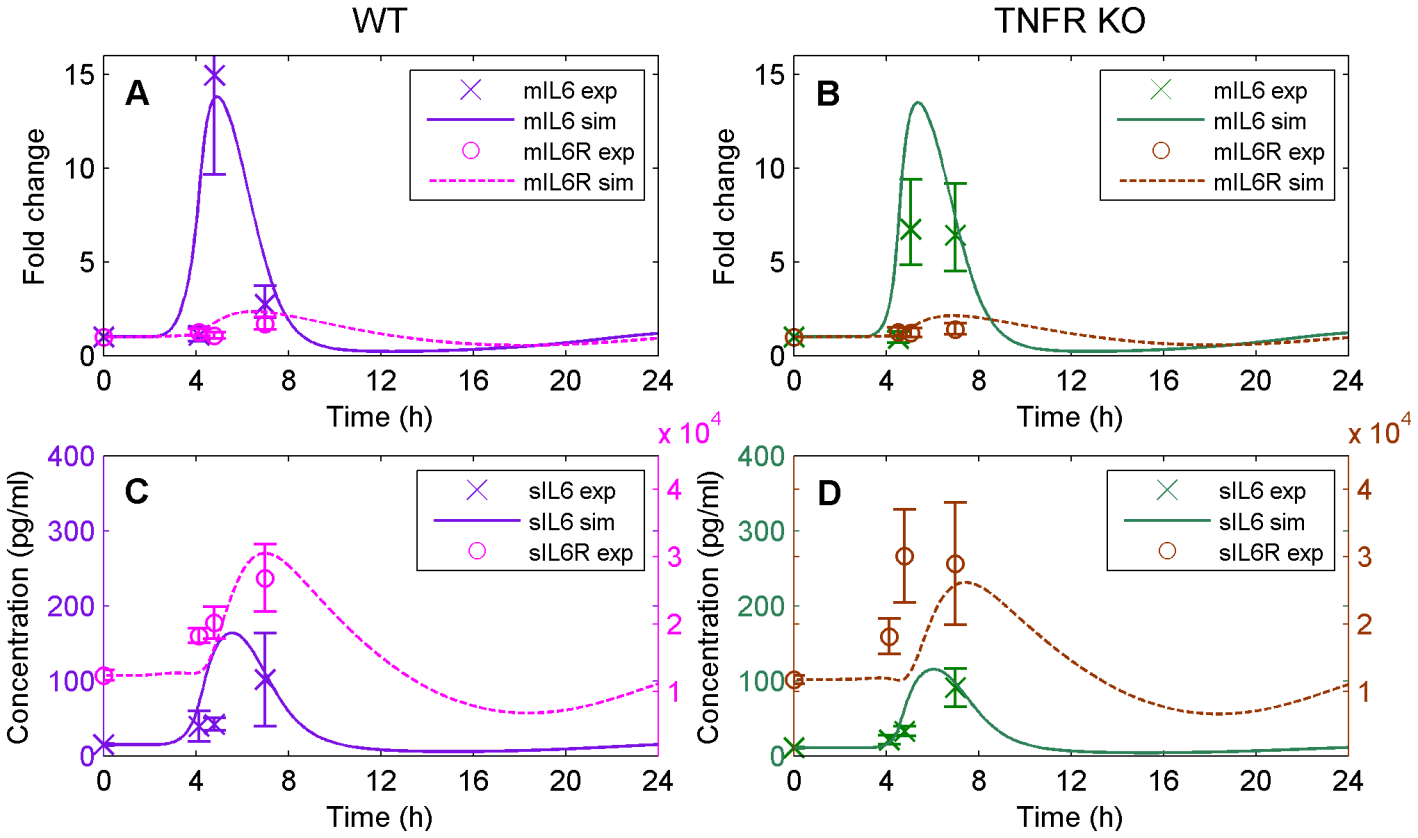
Model simulation (solid and dashed lines) versus experimental data (markers) for the IL-6 family. (A) represents the mRNA accumulation of IL-6 and IL-6R for WT, (B) the mRNA accumulation of IL-6 and IL-6R for TNFR KO (data used for validation only), (C) the plasma concentration of IL-6 and sIL-6R for WT, and (D) the plasma concentration of IL-6 and sIL-6R for TNFR KO (data used for validation only).

Figure 5 shows the dynamics of IL-6 and IL-6R with a structure analogous to Figure 4 (left panels for WT, right panels for TNFR KO; top panels for mRNA accumulation fold change, bottom panels for circulating concentration). WT liver mIL-6R was not significantly affected by HS (dashed line); in contrast, mIL-6 was undetectable at baseline, but ∼13-fold higher than the limit of detection at RTB, which represented the peak time of mRNA accumulation of this cytokine. The model accurately fits the experimental data for both IL-6R and IL-6 (Figure 5A) and model validation showed that the model accurately predicts the TNFR KO response. That is, the model predicts a delay with respect to WT data with maximum mRNA accumulation between RTB and hypothermia (∼6 hours) in contrast to the maximum at RTB experienced by WT animals (∼5 hours, Figures 5A–B). The model predictions for sIL-6 are reasonable with peak levels expected at ∼5–6 hours (Figures 5C–D). On the contrary, sIL-6R responses were dissociated from the time course and magnitude of changes observed in mIL-6R indicating that the liver is not an important source of this circulating protein. As a consequence, the model is not able to accurately predict the experimental changes in sIL-6R for either WT or TNFR KO.

#### IL-10 family

**Figure 6:**
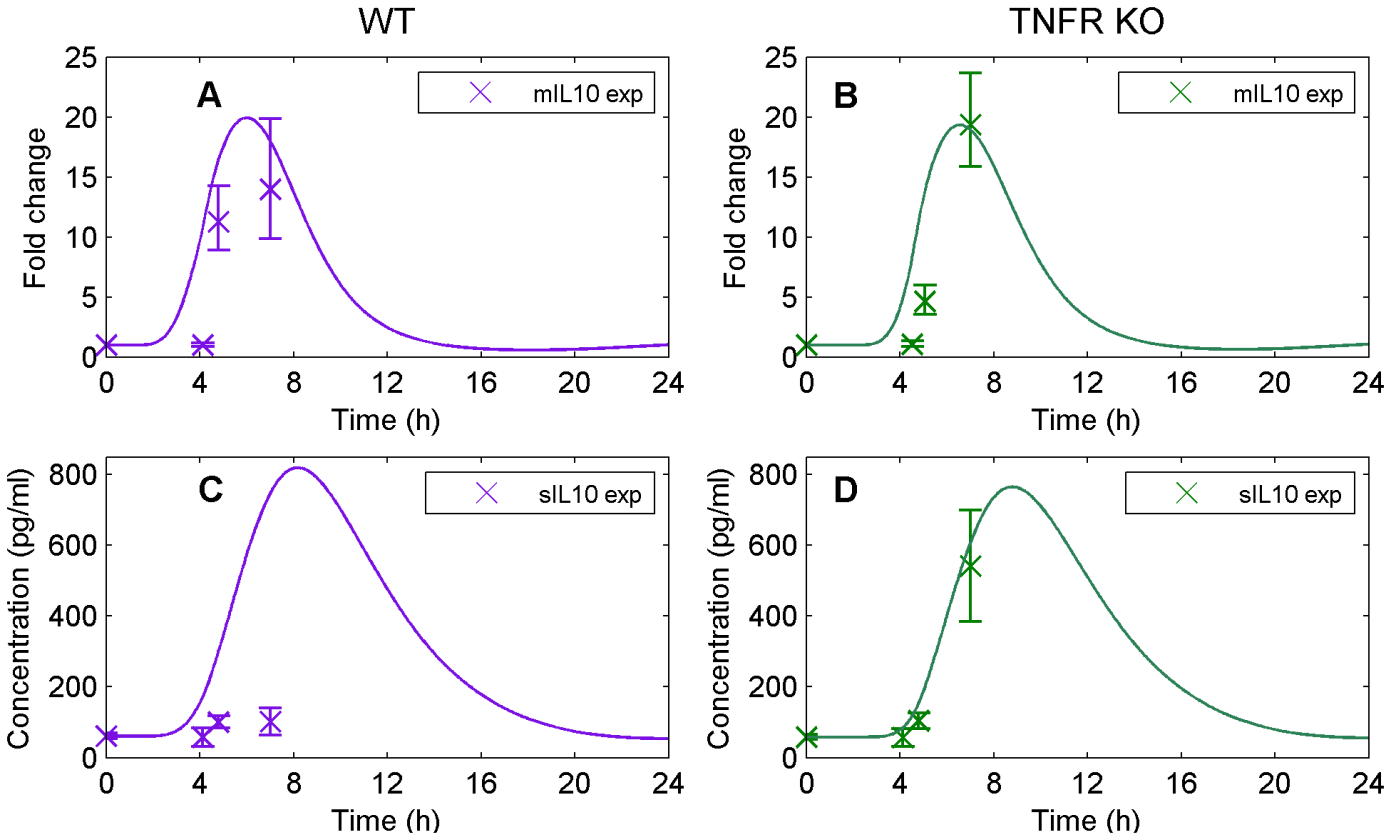
Model simulation (solid and dashed lines) versus experimental data (markers) for IL-10. (A) represents the mRNA accumulation of IL-10 for WT, (B) the mRNA accumulation of IL-10 for TNFR KO (data used for validation only), (C) the plasma concentration of IL-10 for WT, and (D) the plasma concentration of IL-10 for TNFR KO (data used for validation only).

In our experimental mouse study, liver mIL-10 was undetectable at baseline and peaked at hypothermia showing ∼20-fold increase above the limit of detection observed in both WT and TNFR KO mice (see Figures 6A–B); this response was accurately captured by our model. We observed dissociation between mIL-10 and sIL-10 levels that the mathematical model was not able to capture (see Figures 6C–D). That is, liver mIL-10 mRNA accumulation differed between strains at RTB with significantly higher levels observed in WT compared to TNFR KO mice (Table 2 and Figures 6A–B). On the other hand, we have observed significantly lower sIL-10 levels in WT compared to TNFR KO mice at hypothermia (∼150 and ∼500 pg/ml, respectively; [24]), which was not accounted for by our model (Figures 6C–D). This disconnect between predicted and experimental sIL-10 levels might indicate that the mRNA accumulated in the liver is not consistently translated into proteins and/or transported to the plasma, as noted previously for some of the IL-1 and IL-6 family members.

#### TNF-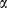 family

**Figure 7:**
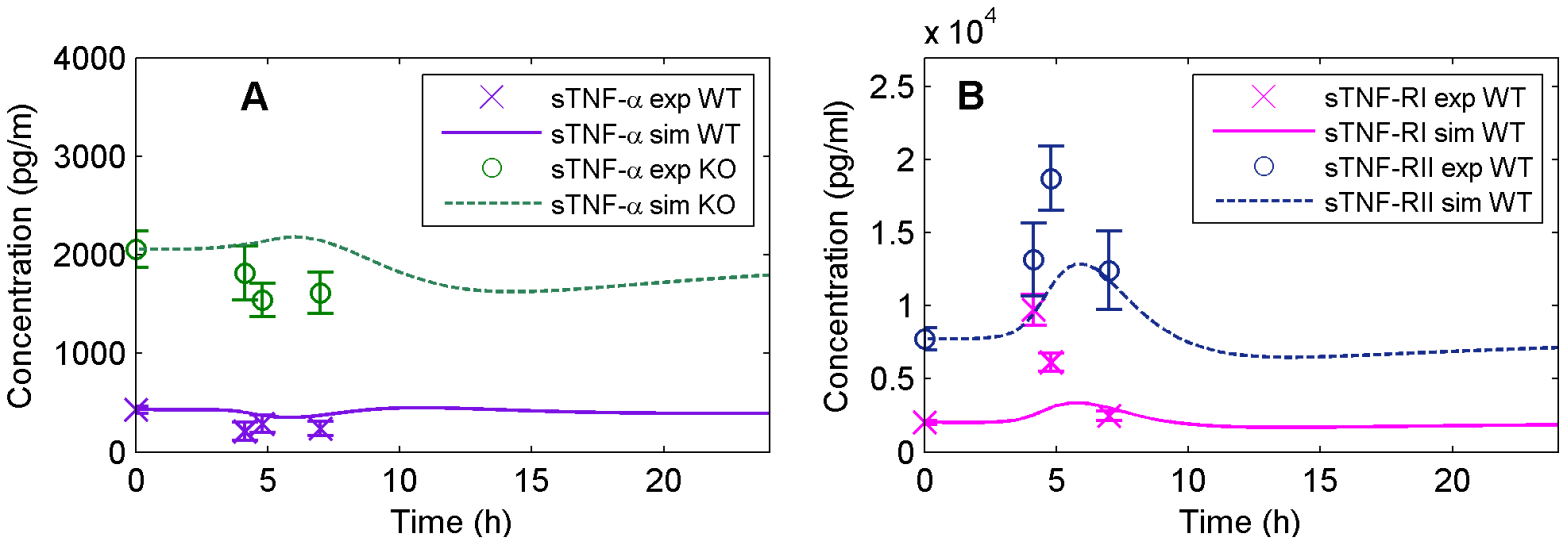
Model simulation (solid and dashed lines) versus experimental data (markers) for the TNF family. (A) represents the plasma concentration of TNF-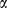 for WT and TNFR KO, and (B) the plasma concentration of the soluble isoforms of receptors TNF-RI and TNF-RII for WT.

Liver mTNF-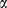 did not show significant change in response to HS in either genotype (Table 2). TNFR KO mice showed ∼6-fold higher sTNF-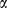 levels than WT mice at baseline and all time points of HS recovery in our model, with no effect of heat exposure on this cytokine (Figure 7, [24]). Our model slightly overestimates the sTNF-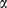 concentration observed for the WT mice and predicted a sTNF-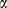 profile for the TNFR KO mice significantly higher than the one observed experimentally (Figure 7A). Liver mTNF-RI and mTNF-RII were not significantly altered by HS; yet, sTNF-RI and sTNF-RII levels showed a significant increase during HS recovery (up to ∼5 fold-change for sTNF-RI at 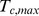; see Figure 7B). The failure of our model to capture this robust change in sTNFRI and II indicates that the liver profile was not accurately reflecting circulating changes in protein; as such, the liver did not appear to be the main source of these soluble receptors. As expected, plasma levels of the sTNF-RI and RII proteins were undetectable at all time points in TNFR KO mice.

### Sensitivity and Correlation Analysis

Despite the use of global optimization techniques, we found several sets of parameters that accurately fit the data, with differences on the order of the precision of the experimental data. Some of the sets lead to indistinguishable model predictions that can be explained by the correlation between parameters, the low sensitivity of some of them, and/or different alternative pathways that the available data are not able to discriminate. The set with the smallest objective function value was selected as the optimal set and used for the simulations depicted in the figures and further analyses.

Local relative sensitivities revealed that, for the optimal parameter set, 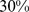 of the parameters account for less than 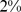 of the information while the 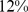 most influential account for more than 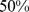 of the total sensitivity. The most influential parameters are those related to HSP transcription, LPS action (initial LPS content in the guts and signaling through TLR4), and IL-6 and IL-1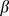 transcriptional activation mediated by AP-1 and NF-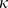
B.

**Figure 8:**
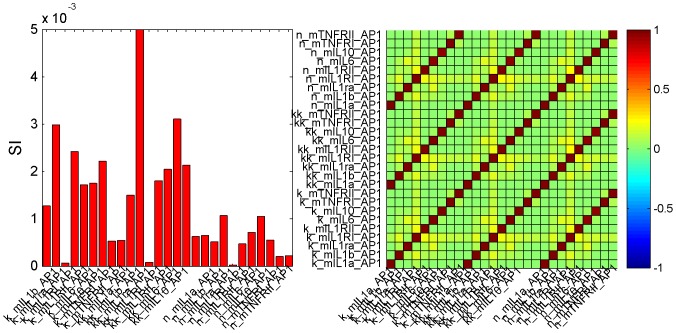
Sensitivity indices and correlation matrix for the Hill function parameters of the AP-1 target genes. SI show that IL-1Ra related parameters are the least influential whereas IL-1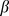 related are the most important. The correlation matrix shows strong correlations among the three parameters involved in each of the Hill equations.

Figure 8 depicts the relative sensitivity indices (SI) and the correlation matrix for the parameters of the Hill functions involved in AP-1 target genes transcriptional activation. SI show that IL-1Ra-related parameters (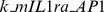, 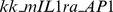, and 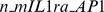) are the least influential whereas IL-1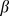-related parameters (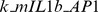, 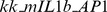, and 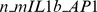) are the most important; this is in agreement with the experimental data, which indicated that IL-1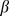 was the main member of the IL-1 family with actions during HS recovery. The correlation matrix shows strong correlation among the three parameters involved in each of the Hill equations (see Eq. 6), which may be the source of some of the identifiability issues.

Strong correlations were also found among the parameters that regulate AP-1 activation by ROS and LPS. This is due to the lack of ROS and LPS experimental data, which makes the distinction between the two main mechanisms for AP-1 activation very difficult.

### *In silico* Experiments

To test the strength and utility of our model for analysis of the heat-induced SIRS, we conducted a series of *in silico* experiments testing the effect of higher heat stress temperature, and knockout of genes that have been implicated in the inflammatory response (*i.e.*, IL-10R). Moreover, an LPS injection experiment similar to the one performed in [21] was simulated and the predicted values for liver mRNA accumulation and plasma concentration for several cytokines were qualitatively compared with the mice data.

### Severity of the Heat Insult

**Figure 9:**
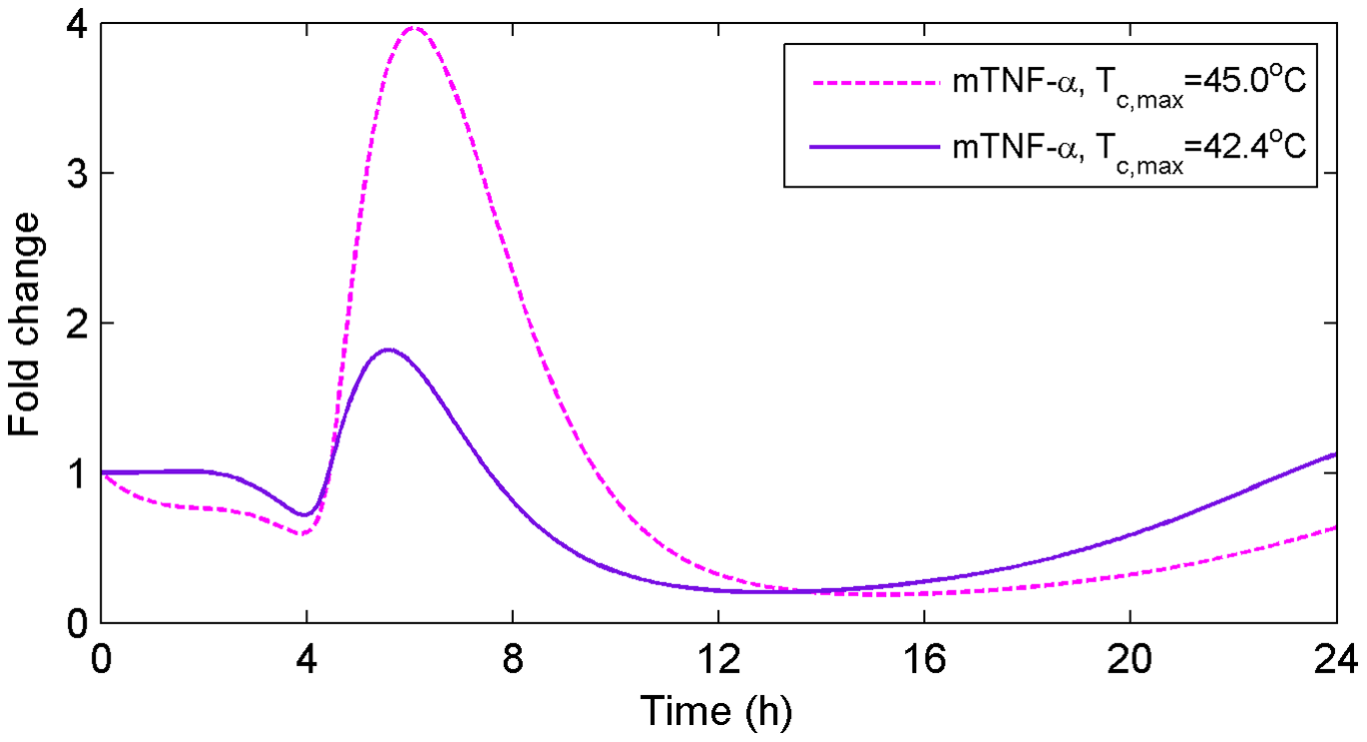
Predicted TNF-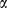 mRNA accumulation for WT mice under different temperature profiles. An *in silico* experiment using a more severe heat insult as an input to the model resulted in increased TNF-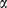 mRNA accumulation levels.

Several studies show divergent results with respect to the circulating TNF-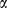 response to HS. Hammami *et al.* [9] were unable to detect elevated circulating TNF-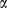 concentrations in 21 HS patients at the time of clinical presentation or shortly after cooling. On the contrary, Bouchama *et al.* [30] observed high circulating TNF-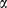 levels at clinical admission in 17 HS patients. It is unclear if incongruity between studies is a complication of differences in experimental procedures, extent of heat injury or other unidentified factors. Several experiments have shown that HSP70 (induced by heat or oral glutamine) inhibits TNF-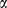 [31]–[34]. Moreover, Shell *et al.* [35] have observed that heat shock inhibits NF-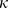
B activation in a dose- and time-dependent manner. An *in silico* experiment using a more severe heat insult as an input to the model resulted in increased TNF-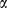 mRNA accumulation levels (Figure 9). In response to 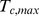 of 42.4°C, which simulates the heat severity experienced by WT and TNFR KO mice in our *in vivo* study, we observed decreased TNF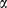 mRNA accumulation throughout HS recovery (Figure 9). Interestingly, no significant changes in TNF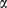 mRNA accumulation were observed during the initial 8 hours of experimentation, which fits our experimental data. Conversely, increasing the 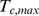 to 45.0°C induced ∼4 fold increase in TNF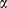 mRNA accumulation within ∼16 hours of recovery. This *in silico* experiment supports the hypothesis in [35] that high or low concentrations of TNF-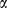 are a function of the duration and severity of the heat insult that might also influence the severity of the damage to the gut epithelial barrier and therefore the release of LPS.

#### IL10-R KO

**Figure 10:**
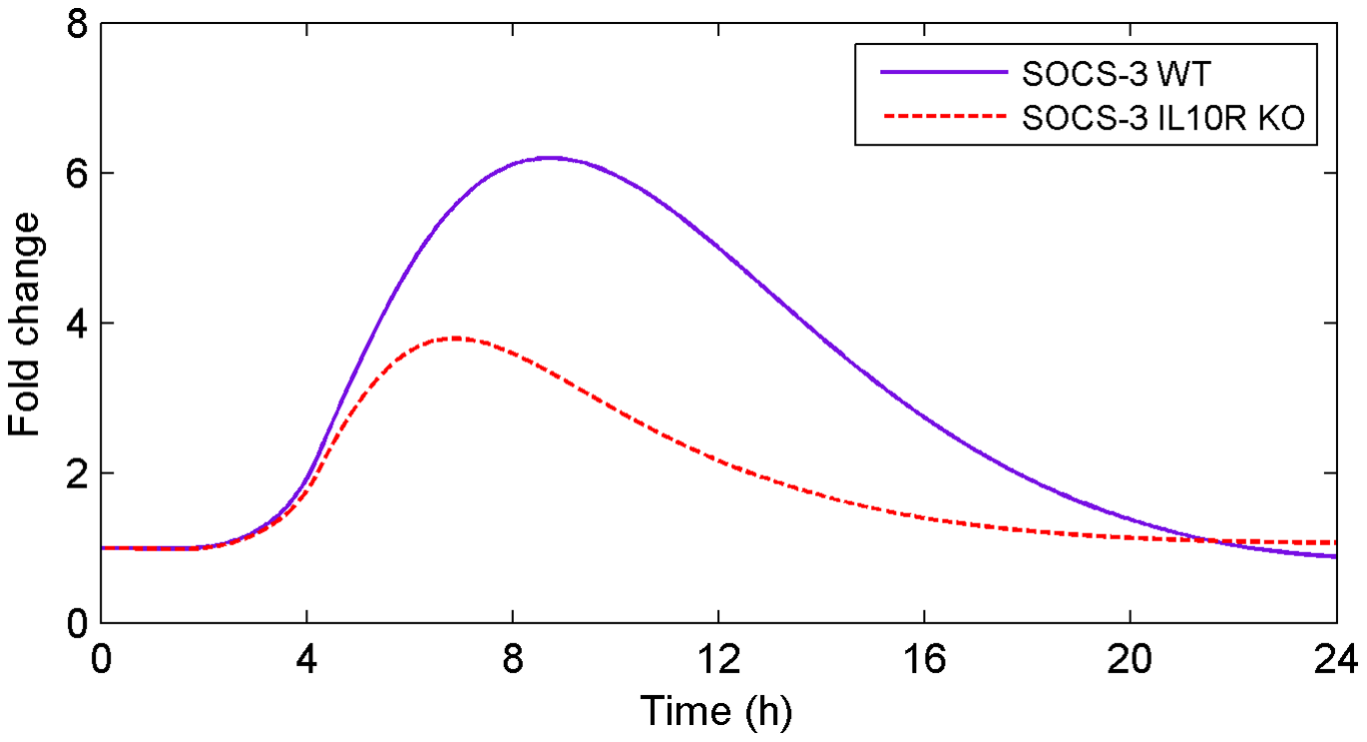
Predicted SOCS-3 mRNA accumulation for WT and IL-10R KO. An *in silico* KO of the IL-10R shows decreased levels of SOCS-3 which may result in an enhanced pro-inflammatory response.

The anti-inflammatory actions of IL-10 are thought to be mediated through the suppressor of cytokine signaling 3 (SOCS-3) pathway. An *in silico* KO of the IL-10R showed that SOCS-3 signaling was significantly decreased compared to the response observed in WT mice (Figure 10). That is, WT mice showed peak accumulation of SOCS-3 mRNA at ∼9 hours; although the time course of this response was similar in the IL-10R KO, SOCS-3 signaling was significantly attenuated compared to the WT condition (Figure 10). These data suggest that SOCS-3 signaling is a downstream target of IL-10 signaling that may regulate the inflammatory response during recovery.

#### LPS injection

**Figure 11:**
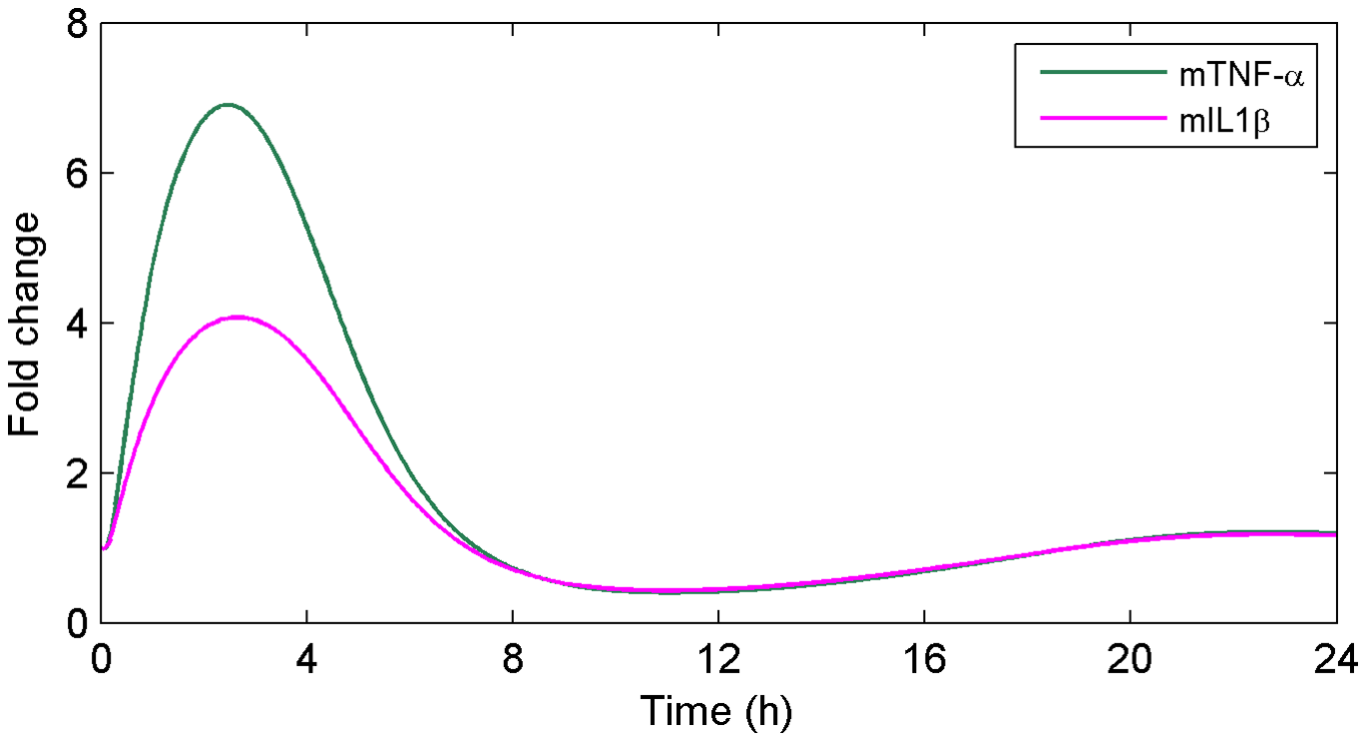
Predicted TNF-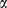 and IL1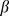 mRNA accumulation after an LPS injection. A simulation of an LPS injection in WT mice shows an immediate inflammatory response with rapid increase of TNF-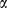 and IL1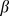 liver mRNA.

In order to assess the validity of our model in a broader context of systemic inflammation we have simulated the experiment described in [21] where WT (C57BL/10) mice were treated with bacterial LPS and liver mRNA levels for TNF-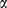 and IL1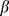, among other cytokines, were measured. The model reproduces the experimental results showing a fast inflammatory response with rapid increase of TNF-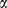 and IL1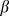 liver mRNA (Figure 11). The level of TNF-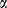 experiences a significantly higher increase than under heat stroke experiment suggesting that LPS might not be playing a crucial role in the SIRS following the heat stroke protocol considered in this work. However, strain differences between studies and the use of only one dose of LPS complicate the interpretation. Future studies will need to be designed to determine the role of LPS as an initiating factor of the SIRS in our heat stroke model.

## Discussion

This is the first study to devise a complex mathematical model of the intra- and extracellular cytokine signaling pathway interactions that are stimulated in response to HS. Literature was thoroughly reviewed and the postulated model integrates relevant biological knowledge and novel experimental data. Tissue damage, DP, LPS, and ROS, stimulated upon 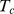 increase, are assumed to initiate a network of HSP and cytokine responses involving HSP70 and four families of cytokines, namely IL-1, IL-6, IL-10, and TNF. Four transcription factors (HSF-1, NF-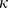
B, AP-1, STAT-3) were considered to be the primary regulators of this network. The biological assumptions made for building this model are supported by solid scientific evidence. However, it has to be noted that the relation among all the species is not exactly known and further experimentation might invalidate some of the assumptions. Moreover, model fitting and sensitivity analysis provide important information about the strength of the postulated interactions giving flexibility to the model for supporting or questioning them.

Hill functions and mass action kinetics were used to build an ODEs model that was calibrated using a set of genomic (liver mRNA accumulation) and proteomic (circulating cytokines and receptors) data by means of global optimization. Despite different factors, as blood volume changes due to dehydration, could influence the value of the model parameters, these effects were considered negligible and all the parameters were considered invariant during the experiment. The model was validated using an independent set of data (not used for calibration) from TNFR KO mice. For both WT and TNFR KO, the model fits the liver qPCR data within the precision of the experimental data; however, for some of the cytokine receptors (e.g. sIL6R, sTNF-RI, sTNF-RII) there is no combination of parameters that accurately fit the liver and plasma data simultaneously since the circulating levels are elevated while the liver mRNA remains unchanged. The deficiencies in the model fit with respect to these soluble cytokine receptors led to this relevant conclusion: molecules newly transcribed and translated in the liver are unlikely to be the primary sources of sIL6R, sTNF-RI, and sTNF-RII in the circulation during HS. Since the model was built based on this hypothesis, this fitting mismatch can only be overcome by changing the model structure and including other sources of circulating cytokines. Although changes of clearance and/or degradation rates during HS could eventually explain this divergence, the most plausible hypothesis is that other organs/cell types (circulating macrophages, splenocytes), which we plan to integrate into a more comprehensive model in the future, are also contributing to the circulating cytokine milieu. As we move forward with this modeling effort, these insights will be critically important for us to identify the sources of circulating cytokines and potential therapeutic targets that can be tested physiologically in our mouse model. Moreover, these findings motivate the development of a multi-modular model by systematic incorporation of other relevant peripheral organs, such as the spleen and the brain, and ultimately the entire body.

For some of the species (e.g. IL-1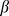, IL-1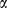, IL-10), changes in liver mRNA accumulation are not reflected in the circulating protein levels. Although our computational model is able to capture these dynamics, the low values estimated for the translation/transport parameters associated with these cytokines suggest that the mRNA is not consistently translated into proteins, the proteins generated in the liver are not extensively released into the blood stream, and/or they present a long delay not captured within the time frame of our experimental protocol. To discriminate between these scenarios, studies on protein translation including liver protein concentration assays and protein tagging should be performed.

NF-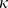
B remains practically unchanged in the liver of WT and TNFR KO mice under the experimental HS protocol used in this study. These findings are in agreement with those of [36] that showed that NF-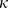
B DNA binding activity did not change in response to heat stress in rats; yet, they are somewhat surprising since NF-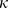
B has been implicated as the main TF that regulates cytokine mRNA accumulation during inflammation [37]. Moreover, both TNF-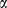 liver mRNA accumulation and plasma concentrations (activated by NF-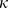
B) were not affected by the heat insult in contrast to other studies that reported elevated concentrations of this cytokine after HS [30]. Although these discrepancies between studies may be partly explained by species differences in HS responses, a more plausible explanation is that the highly elevated mRNA accumulation of HSP70 inhibited the translocation of NF-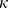
B to the nucleus in a dose- and time-dependent manner [35]. We hypothesize that activation of NF-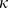
B and increased concentration of TNF-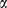 depend on the duration and severity of the heat insult. An *in silico* experiment with a more severe heat insult illustrates that our model supports this hypothesis with a molecular mechanism (inhibition of NF-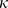
B by HSP70) capable of explaining the different behaviors, encompassing the apparently contradictory results from the literature.

AP-1 is revealed here as the main transcription factor activated in the liver during HS recovery and it appears to be responsible for the high expression of many pro-inflammatory cytokines (*i.e.* IL-1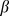, IL-6, and IL-10) in spite of the low activation of NF-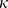
B. The fact that JUN and FOS liver mRNA accumulation is lower in TNFR KO mice at baseline indicates that the differences in TNFR KO responses are not only due to differences in heating and cooling but a direct effect of the lack of TNF signaling. It is intriguing to speculate that lower JUN and FOS mRNA accumulation may be a direct consequence of the absence of a TNF autocrine cascade effect on AP-1 activation, which has been shown to be triggered in hepatocytes in response to TNF [38]. IL-1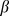, IL-1RI, IL-6, and IL-6R also presented lower mRNA accumulation in normothermic conditions for TNFR KO, which can be explained by the lower activation of AP-1. However, we reject the hypothesis that the changes in liver gene expression in our model were only related to the TNF autocrine cascade since we failed to detect changes in liver TNF mRNA accumulation in WT mice at any time point of HS recovery. Rather, our data suggest that delayed liver gene expression was due to a combination of factors that include: 1) lower initial (baseline) expression; 2) indirect effect of other genes that activate liver AP-1 that also showed a delayed response (*i.e.* IL-6, IL-1); 3) lower core temperature of TNFR KO mice during heat exposure. Unfortunately, it was not possible to normalize the heating responses between genotypes since the absence of TNF signaling induced a downward shift in the temperature set point, thus causing the KO mice to regulate 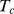 and metabolic rate differentially from WT mice [24].

AP-1 may deactivate the heat shock response during stress recovery by hyperphosphorylating HSF-1 to inhibit its function [39], [40]; hence, robust activation of AP-1 (e.g. due to an excess of LPS) could lead to considerably decreased HSP70 gene expression in response to HS. Since HSP70 is concomitantly inhibiting the activation of NF-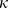
B, HSP70 downregulation could exacerbate the situation leading to a lethal concentration of proinflammatory NF-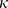
B dependent genes, such as TNF-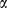. Therefore, AP-1 and its related cofactor genes (Jun and Fos families) are revealed here as promising targets for future therapeutic intervention to accelerate HS recovery. In contrast, HSPs exert an anti-inflammatory effect by inhibiting the translocation of NF-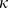
B to the nucleus; therefore, it is expected that drug-induced increases in HSP70 gene expression prior to or following HS collapse may attenuate the heat-induced SIRS in the liver and perhaps other organ systems.

An important outcome of our predictive model is the identification of a differential time course of circulating cytokine responses in TNFR KO mice compared to their WT controls. This is an important aspect of our model as it identified unique time windows that should be considered in future analyses of the circulating biomarkers that may be mediating damage. Furthermore, it suggests that alterations of the cytokine balance (*i.e.*, WT *vs.* TNF KO) may skew the cytokine milieu towards a pro- or anti-inflammatory phenotype that alters the time course of progression of the SIRS. Future studies in our mouse HS model that target different time points or core temperatures for cytokine analysis will be instrumental in defining the rapid changes in cytokine production that mediate changes in our model and may have been missed in our current analysis.

The uneven distribution of the parameter sensitivities found in this study reinforces Gutenkunst *et al.* [41] who concluded, after testing several systems biology models, that “sloppy” spectra of parameter sensitivities, *i.e.* with eigenvalues roughly evenly distributed over many decades, are universal in systems biology models. This property may explain the difficulty of extracting precise parameter estimates from collective fits and reinforces the need for establishing a parameter ranking. For that reason, in this work the parameter estimation was done in two stages; first we focused on the most influential group of parameters, whereas the less important group was fitted in a second stage. Despite the sequential parameter identification and the use of global optimization techniques, the identifiability analysis revealed a number of difficulties with estimating a unique value for the parameters. Although model reduction could be attempted at this stage, we preferred to analyze the detailed mechanistic model and exploit the identifiability deficiencies encountered for planning future experiments aiming to obtain a complete picture of the SIRS ensuing during HS recovery. In particular, further experimentation that assesses changes in circulating LPS concentrations or the effect of neutralizing its effects (with antibiotics or gut sterilization treatments) will be important to improve identifiability and discriminate among ROS and LPS overlapping mechanisms. In this direction, the work recently published [42] showing increased mortality of TLR4 KO mice under heat stroke suggests that LPS might not play a significant pathogenic role.

In summary, the present work provides new insights into the molecular mechanisms underlying the complex etiology of HS and defines a framework that supports *in silico* exploration of cytokine signaling pathways in response to HS. This type of modeling framework not only aids in the development of new methods that will reduce the need for timely and costly animal experiments, but also increases the rapidity and accuracy with which novel pharmacologic intervention and/or treatments are identified to treat this debilitating illness. However, it should be noted that this study is exploratory and a larger scale study is needed to confirm the results in the future.

## Methods

### Ethics Statement

In conducting the research described in this report, the investigators adhered to the “Guide for Care and Use of Laboratory Animals” as prepared by the Committee on Care and Use of Laboratory Animals of the Institute of Laboratory Animal Resources, National Research Council. The protocol was approved by the Scientific Review Committee and the Institutional Animal Care and Use Committee “US Army Research Institute of Environmental Medicine Institutional Animal Care and Use Committee” (Permit Number: A09-02).

### Description of the Data

Details of the HS protocol are described elsewhere [43]. In this study, conscious, unrestrained male B6129F_2_ (wild-type; WT) and TNF-RI/R-II knockout (TNFR KO) mice (originally at a normal housing temperature of 25±0.2°C) were exposed to an ambient temperature (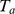) of 39.5±0.2°C in an incubator, in the absence of food and water, until a 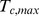 of 42.4°C was attained. Following removal from the heat at 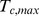, food and water were provided ad libitum during undisturbed recovery at 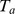 of 25±2°C.

Prolonged heat exposure induces thermoregulatory changes consisting of hyperthermia in response to direct heat exposure, and a biphasic response characterized by hypothermia which develops within ∼3 hours of recovery [5], [18]. The initiation of the SIRS is thought to occur within the time frame from 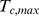 to hypothermia, during which endotoxin is thought to leak across ischemic damaged gut membranes into the portal circulation [5], [6]. To identify the inflammatory pathways mediating the early stages of the SIRS, mice were assigned to one of the following groups for blood and tissue collection: 1) baseline (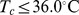; immediately prior to heat stress), 2) 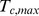 (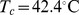), 3) return to baseline (RTB; first 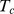 value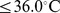 during cooling), or 4) hypothermia depth (lowest 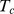 value with cooling rate of 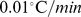 during recovery). Control mice were tested at 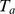 of 25°C at their original cage location and not exposed to the incubator environment. A HS group was assigned to each sampling time point, except baseline, which was represented by one control (nonheated) group for each genotype. Control groups were included at each time point to examine circadian influences on the measured variables in the absence of HS. Group sizes for each sampling time point were as follows: baseline: WT, n = 10; TNFR KO, n = 8; 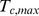: WT control, n = 7; TNFR KO control, n = 6; WT heat, n = 7; TNFR KO heat, n = 6; RTB: WT control, n = 8; TNFR KO control, n = 8; WT heat, n = 8; TNFR KO heat, n = 9; hypothermia: WT control, n = 6; TNFR KO control, n = 8; WT heat, n = 8; TNFR KO heat, n = 8. Note that owing to the need for sacrificing mice to obtain biological samples, data at different time points correspond to different mice, which introduced additional noise due to the inherent variability among animals. Furthermore, this experimental design precluded an analysis of survival rates in this study.

#### Core temperature and dehydration


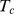
 was continuously monitored at 1-min intervals using an intraperitoneally implanted battery-free radiotelemetry transmitter (model G2 Emitter, Mini Mitter Co., Inc., Bend, OR) in conscious, unrestrained mice. Mice were implanted with a transmitter device at least 2 weeks prior to experimentation to ensure full recovery prior to sample collection. The level of dehydration was estimated by the difference in body weight before and after heat exposure determined on a top-loading balance (0.1 g) and corrected for transmitter weight; however, we did not account for urine of feces loss [24]. Body weight at the beginning of the experiment was similar between genotypes at ∼27 g.

In order to use mean values for the genomic and proteomic data obtained from different animals, an average of the 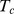 profile for each of the mouse strains was used as input to the system (see Figure 2). The sampling points for data collection were based on mouse 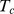 responses; therefore, each data point corresponding to maximum core temperature (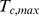), RTB, and hypothermia depth was collected at a different time point of recovery with respect to the onset of heat exposure (*i.e.*, mice heat and cool at different rates despite similar body weights prior to experimentation). For this reason, averaging along the time axis would lead to misleading results in terms of temperature values (see Supporting Information S1); thus averaging along the temperature axis was preferred. To this aim, we proceeded in four steps: i) filtered the individual curves, ii) divided them into increasing and decreasing temperature sections (to ensure bijective functions), iii) inversely mapped by interpolating the time values for a set of temperatures, and iv) computed the mean with respect to the temperature for the interpolated times. Figure 2 shows the resultant 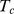 profiles that were used as an input to the mathematical model. Circadian 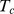, time to reach 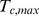, thermal load, cooling time to baseline, and time to hypothermia were analyzed using one-way analysis of variance (ANOVA) and the Holm-Sidak method for post-hoc comparisons, setting the significance at 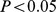.

Previous experiments where 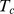 was monitored throughout 72 hours of recovery [43] showed that after hypothermia (∼6 hours) 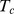 remains at normothermia or lower until ∼24–32 hours when a fever-like increase is observed. The model is based on the assumption that elevated 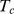 is the initiator of the inflammatory response by means of DP, LPS, and ROS; therefore, although temperature measurements after hyperthermia are not available for this experiment, we assumed that between 6 and 24 hours no LPS is released and the production of DP and ROS is not increased from its basal level.

#### Liver mRNA accumulation

The threshold cycle (Ct) for a variety of genes implicated in the response to heat stroke was determined in liver tissue by means of qPCR (quantitative polymerase chain reaction). The Ct for each gene was defined as the PCR cycle at which the emitted fluorescence signal was greater than the background fluorescence level [44]. Amplification was determined to be detectable if 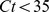. Changes in mRNA accumulation were calculated as fold-change relative to the controls matched to the same sampling point using the 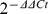 method [45], where
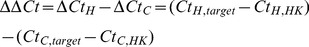
(1)being 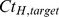, 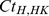, 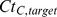, and 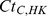 the Ct of the target and housekeeping (HK) genes under HS and control conditions, respectively. qPCR results are presented with asymmetric standard error bars in the figures due to the exponential relation between Ct and fold-change. The standard deviation, propagating the error of both control and experimental groups, is given by
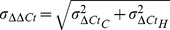
(2)where 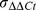 represents the standard deviation of 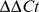; 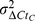 and 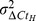 are the standard deviation of the difference between target and HK gene threshold cycles for mice under control and heat stress conditions, respectively.

Liver mRNA accumulation was measured for HSP70, NF-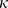
B family members (NF-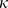
B1, NF-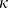
B2, RelA, RelB, c-Rel, and I
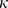
B), Jun and Fos genes (related to AP-1), and the following cytokines and cytokine receptors: IL-1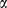, IL-1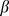, IL-1RI, IL-1RII, IL-6, IL-6R, gp130, IL-10, TNF-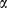, TNF-RI, and TNF-RII.

#### Plasma cytokines data

Plasma level of cytokines and soluble receptors was determined on duplicate samples using the FlowMetrix™ System (Luminex, Austin, TX), which permits the simultaneous quantitation of multiple cytokines. The FlowMetrix System is a “Multiplexed Fluorescent Bead-Based Immunoassay”, with the kits used in this study being specific for mouse cytokines. Sensitivities of the cytokine and soluble cytokine receptor assays were ∼2 and ∼20 pg/ml, respectively. The plasma cytokine and soluble receptor data used to calibrate the model correspond to IL-1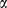, IL-1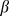, sIL-1RI, sIL-1RII, IL-6, sIL-6R, sgp130, IL-10, TNF-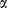, sTNF-RI, and sTNF-RII. The experimental data of circulating cytokines, including control and HS measurements, were analyzed to detect outliers and significant changes between data collected at different sampling points. The error bars in the figures represent the standard error of the measures (mean
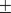
SE). The original data from this analysis are presented elsewhere [24].

### Modeling and Biological Assumptions

For the sake of parsimony, we used first-order kinetics to model activation of the TFs [46]. Therefore, the rate of activation of the TFs is proportional to the concentration of the activator 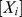 times the concentration of the inactive TF, denoted by 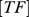:
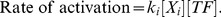
(3)


Moreover, we assumed that the total number of active, 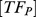, and inactive forms of TF is conserved:
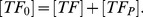
(4)


The rate of change of 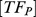 is given by the balance between its activation rate by all the possible activators (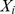), the deactivation due to all the inhibitors (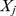), and an autonomous deactivation at a rate 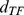:
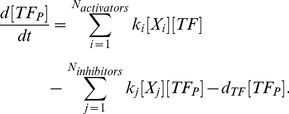
(5)


Since the signaling networks are usually very complex and information is lacking from our study regarding the intermediate states, we made some simplifications through lumped states. The activators represent the ligand-receptor complexes or other substances as DP that initiate and/or potentiate the signaling cascade.

The transcriptional activation was modeled using Hill functions and mRNA degradation rate was assumed to be linear:
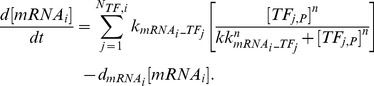
(6)


Mass action kinetics was used for describing the translation of mRNA into proteins (cytokines and cytokine receptors) and complex formation:
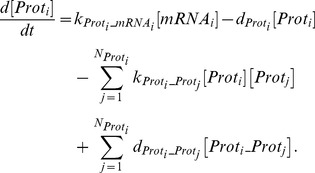
(7)


Note that 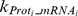 are not pure translation rates since they also account for the transport of the cytokines outside the cell.

The soluble receptors involved in this model (sIL-1RI, sIL-1RII, sIL-6R, sgp130, sTNF-RI, and sTNF-RII) are produced mainly by proteolytic cleavage of the extracellular domains of their analogous membrane bound forms [47]–[49]. Therefore, their concentration was modeled as follows:
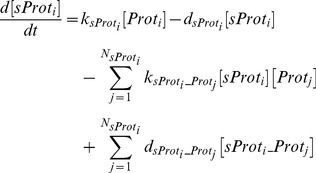
(8)and the corresponding expression for the analogous cell-surface receptor is given by Eq. (7) plus an additional term accounting for the conversion into the soluble form (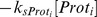).

Without heat the model is assumed to be at steady state (circadian effects were not statistically significant):
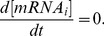
(9)


We assumed a normalized expression rate for all genes and active concentration of the transcription factors to be equal to one (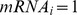 and 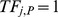). Therefore, we computed degradation rates as:
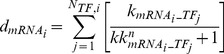
(10)and significantly decreased the number of parameters to be estimated (from 217 to 130).

#### LPS

Different types of environmental stressors, including heat, can cause increased intestinal permeability that facilitates endotoxin leakage across ischemic-damaged gut epithelial membranes and induces local and/or systemic inflammatory reactions [50]. The presence of circulating endotoxin in some HS patients is thought to be due to leakage following gastrointestinal barrier disruption, but may also be related to liver damage [51]. Since the liver is one of the major sites of endotoxin clearance, damage to this organ may result in increased susceptibility of mammals to the heat-induced SIRS mediated by endotoxin.

Su *et al.* [52] performed *in vivo* analyses of physiologically relevant barrier dysfunction and determined mouse jejunal permeability by measuring the paracellular bovine serum albumin (BSA) flux under standard conditions as 0.18 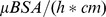. Lambert *et al.* [12] and Dokadny *et al.* [53] showed significant time- and temperature-dependent increases in gastrointestinal permeability following both modest and severe heat insults. Although several cytokines and other factors are known to regulate the permeability of the gut epithelial barrier [54], in this work we only considered permeability influenced by temperature, assuming an exponential increase from the basal value (0.18 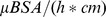) when 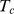 is above 41°C. Increased gut epithelial barrier permeability at this core temperature resulted in the release of LPS which was modeled using mass action kinetics with the following equation:
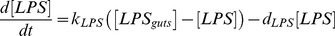
(11)where 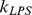 represents the gut epithelial barrier permeability and increases with the core temperature, 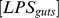 is the initial concentration of LPS in the gastrointestinal tract of the mouse and 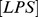 is the concentration of LPS in the circulation. LPS acts as the prototypical endotoxin because it binds the Toll-like receptor 4 (TLR4) to induce a signal transduction cascade that ultimately triggers essential signaling modules resulting in activation of the transcription factors NF-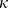
B and AP-1 [55] following equation (5).

#### Oxidative Stress

Heat stress increased reactive oxygen species (ROS) generation and free radical-mediated splanchnic injury of young rats [56]. Furthermore, reactive oxygen and nitrogen species production is thought to be involved in regulation of redox-sensitive transcription factors, such as AP-1 and NF-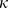
B, that mediate the expression of inflammatory mediators such as cytokines, chemokines, and adhesion molecules [57]. Moreover, oxidative stress impairs the heat stress response and delays unfolded protein recovery and function, which may compromise protective functions of some protein pathways during heat stress [58].

Zhang *et al.* [36] observed a small, transient increase in hepatic oxidative damage in young rats that experienced minimal liver damage under heat stress; however, this response was accompanied by a sharp and significant elevation of AP-1 DNA binding activity suggesting that this TF is mediating inflammatory changes in the liver during recovery. In contrast, NF-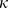
B DNA binding activity did not change following heat stress indicating that NF-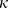
B might not be a main regulator of cytokine gene expression under these conditions. It should be noted that the results of this study cannot be examined solely in the context of heat stress because the research design did not control for dehydration that was experienced during heat exposure, which is an adverse physiological response that is known to increase oxidative and cellular stress [59]. In order to encompass these experimental studies, ROS are generated in our model following zero-order kinetics with varying 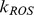 and degraded following first-order kinetics:
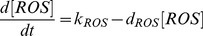
(12)where 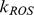 is a function of the temperature and the dehydration level. ROS activates AP-1 transcription following equation (5) and inhibits HSF as described below in equation (18).

#### Denatured proteins, HSPs, and HSF-1

HSPs are important modulators of both anti-inflammatory and pro-inflammatory responses. Asea *et al.* [60] introduced the term chaperokine, to describe the dual role of most HSPs as chaperones with an intracellular cytoprotective/antiapoptotic function and cytokines with an extracellular immunogenic function. In non-stressed conditions, HSPs function as molecular chaperones by maintaining protein conformation and facilitating transport throughout the cell’s various compartments. Under environmental stress conditions, the structural integrity of cellular proteins is compromised and binding of HSPs to these damaged proteins prevents aggregation and supports refolding.

Hyperthermic temperatures cause accumulation of denatured proteins and exposure of their hydrophobic domains which stimulates the expression of HSP genes (especially HSP70, the most highly heat-inducible member) through activation of HSF-1 [61]. The onset temperature for this response is usually ∼40°C, but some transitions may extend as low as 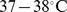 [62]; therefore, we utilized the equation in [63] to calculate the fractional protein denaturation, 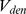, as a function of the temperature in the range of 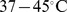:
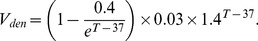
(13)


The amount of protein that denatures per unit of time is obtained by multiplying 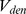 by the amount of native proteins (P):
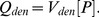
(14)


The native proteins are obtained by subtracting the denatured proteins (DP) from the total amount of native proteins susceptible to denaturation in the range of 37–45°C (P0), approximated as 10% of the total amount of proteins in the cell:
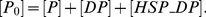
(15)


Consistent with [63], the amount of denatured proteins which complex with HSP70 is determined by mass balance in the present model. In contrast to the other mass balances in the model in which there is an interaction between two substances to form a complex, in this mass balance free HSP70 interacts with both the denatured and the native proteins: free HSP70 (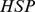) binds to free DP (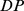), reducing the amount of free DP in the cell, while the released (renatured) proteins are added to the pool of native proteins in the cell. The complexation is described by the following relation:
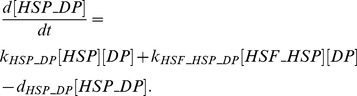
(16)


The first term describes the forming of the HSP_DP complexes while the last term describes the dissociation of the complexes into renatured proteins. The amount of free denatured proteins, DP, is obtained from the amount of denaturing proteins per unit of time, 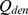, minus the amount of DP’s complexed with HSP70:
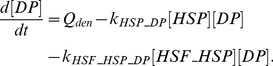
(17)


Before heat-induced activation, HSF-1 exists as a monomer localized to the cytoplasm with much of it forming a heterodimer complex with HSP70. HSP70 preferentially binds to denatured proteins; therefore, it has been postulated that activation of HSF-1 may occur as a result of competitive release of this TF from the HSF1–HSP70 complex when the concentration of denatured cytoplasmic proteins increases as a result of heat shock [26], [64]. As a result, the equation describing HSF-1 dynamics reads:
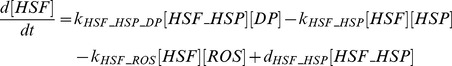
(18)which is equivalent to eq. (5) being 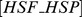 the inactive state of HSF, [DP] the activator, and [ROS] the inhibitor.

HSP mRNA (mHSP) transcription was modeled using eq. (6) where the only TF is HSF. The Hill coefficient for HSF-1 was taken from [65] as 1.5. The intermediate value for the apparent Hill coefficient of between 1 and 2 suggests that one HSF trimer may bind stably, and a second may bind only weakly or partially. mHSP is then translated into HSPs through eq. (7), which exert an anti-inflammatory effect by inhibiting translocation of NF-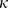
B to the nucleus and preventing expression of inflammatory mediators in a dose- and time-dependent manner [35]. Moreover, the crucial indirect role of HSPs in maintaining gut epithelial barrier integrity suggests an important anti-inflammatory effect by attenuating endotoxin leakage into the circulation, which should mitigate the heat-induced SIRS [66].

#### NF-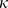
B

NF-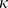
B is a ubiquitous TF of particular importance in immune and inflammatory responses. The larger NF-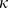
B family is composed of two subfamilies: the NF-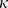
B subfamily that includes NF-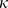
B1 (p50/p105) and NF-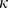
B2 (p52/p100) proteins, and the Rel subfamily that includes RelA (p65), RelB, and c-Rel. All these structurally-related proteins can form homodimers or heterodimers (except for RelB, which only forms heterodimers). NF-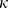
B belongs to the class of “rapid-acting” primary TFs, which are present in cells in an inactive state and do not require protein synthesis to be activated. Rel/NF-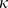
B transcription complexes are present in a latent, inactive state in the cytoplasm due to binding to the inhibitor I
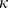
B. A variety of stimuli, namely LPS through binding to TLR4, rapidly activate Rel/NF-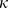
B transcription complexes by releasing the TFs from inhibitor binding, which allows translocation to the nucleus for binding to 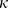
B sites that regulate the expression of many genes [67]. The pro-inflammatory cytokines IL-1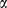, IL-1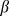, and TNF-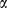 experience reciprocal activation with NF-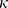
B [68]. NF-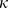
B also regulates the expression of additional genes encoding proteins involved in the heat shock response, including IL-1Ra, IL-1RII, IL-6, IL-6R, gp-130, IL-10, IL-10R, and TNF-RII [27], [68]–[70].

The development of mathematical models of NF-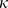
B signaling, tightly linked to experimental results, has been instrumental in unraveling the forms of regulation in NF-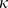
B signaling and their underlying molecular mechanisms [71], [72]. Unfortunately, due to the lack of data on nuclear translocation, we were not able to incorporate details on NF-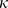
B activation into our model; thus, we assumed first order kinetics (Eq. 5) treating the aforementioned TF as a lumped state. The apparent Hill coefficient for the transcriptional activation of downstream genes was taken from the literature and assumed to have a value of 2 [73], [74].

#### AP-1

AP-1 is another essential TF that regulates inflammatory and immune genes. AP-1 is a group of structurally and functionally related members of the Jun and Fos protein families. Jun proteins exist as homo- and heterodimers whereas Fos proteins, which cannot homodimerize, form stable heterodimers with Jun proteins, thereby enhancing their DNA-binding activity. AP-1 activity is regulated by a broad range of physiological and pathological stimuli, including cytokines, growth factors, stress signals, infectious agents, and oncogenic stimuli. Regulation of net AP-1 activity can be achieved through changes in transcription of genes encoding AP-1 subunits, stability of their mRNAs, posttranslational processing and turnover of pre-existing or newly synthesized AP-1 subunits, and specific interactions between AP-1 proteins and other TFs and cofactors [75].

Developing a detailed model for AP-1 activation is out of the scope of this work; therefore, we assumed first order kinetics (Eq. 5) considering AP-1 as a lumped state activated by LPS through TLR4, IL-1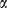, IL-1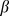, IL-6, and ROS. Among the genes involved in the cytokine network with HS, nine are known to be AP-1 target genes: IL-1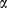, IL-1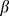, IL-1Ra, IL-1RI, IL-1RII, IL-6, IL-10, TNF-RI, TNF-RII [26], [76], [77]. Transcriptional activation of these genes was modeled using Eq. (6) with a Hill coefficient of 2 (AP-1 Hill coefficient was found to vary between 1.6 and 2.6 [78]).

#### STAT-3

STAT-3 is a TF with fundamental importance for cytokine-mediated induction of acute-phase response genes and is a key regulator of gene expression in response to IL-10 and glycoprotein 130 (gp130) family cytokine signaling (e.g., IL-6) [79], [80]. IL-10 acts as a more potent anti-inflammatory cytokine than IL-6, although both cytokines activate STAT-3. Cytokine binding to cell surface receptors induces receptor-associated Janus tyrosine kinase 1 (JAK1) activation leading to phosphorylation of a single tyrosine residue in the STAT-3 molecule. Phosphorylation results in STAT-3 dimerization and nuclear entry for binding to specific DNA sequences in the promoter regions of target genes [80].

STAT-3 is thought to upregulate the following proteins involved in the cytokine network during heat stroke: IL-1R1, IL-6R, gp130, IL-10, and suppressor of cytokine signaling 3 (SOCS-3) [70], [80]–[82]. The initial activation of STAT-3 by IL-10 or IL-6 (Eq. 5) precedes SOCS-3 gene expression (Eq. 6) with subsequent inhibitory effects of SOCS-3 on gp130/IL-6 signaling pathways [83].

#### IL-1 family

IL-1 is an important mediator of inflammation and tissue damage in multiple organs. The IL-1 family consists of two agonists (IL-1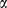 and IL-1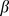), two receptors (biologically active IL-1RI and inert IL-1RII), and a specific receptor antagonist (IL-1Ra). IL-1 signals through a single receptor (IL-1RI) that binds IL-1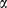 and IL-1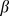 with equal affinity, while IL-1RII preferentially binds IL-1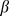. IL-1Ra prevents the association of the IL-1 ligands with the IL-1Rs; thus, the balance between IL-1 and IL-1Ra in local tissues plays an important role in susceptibility and severity of many disease conditions [27], [84]. IL-1 signaling activates NF-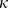
B and AP-1 to induce the expression of many genes [85].

Both IL-1RI and IL-1RII have soluble isoforms (sIL-1RI and sIL-1RII) that are generated by proteolytic cleavage of their extracellular domains modeled by Eq. (8). These sIL-1Rs bind and inhibit IL-1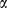 and IL-1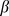 signaling. Because IL-1Ra binds avidly to both the membrane-bound and soluble forms of IL-1RI, sIL-1RI is likely the principal antagonist of IL-lRa. Therefore, although sIL-1RII antagonizes the action of IL-l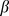, sIL-1RI indirectly may serve to facilitate the activity of IL-l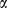 and IL-1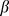 by binding to IL-lRa [86].

#### IL-6 family

Circulating IL-6 shows the highest correlation with mortality and neurologic symptoms in HS patients, suggesting this cytokine may be an important therapeutic target for prevention/treatment strategies. However, IL-6 KO mice showed higher mortality rates than WT mice indicating that IL-6 also has protective, anti-inflammatory actions that are critical for survival [18]. IL-6 is an important mediator of the acute-phase response to injury and infection and induces its cellular actions through two signaling pathways that have opposing actions [87]. In the “classic” (anti-inflammatory) pathway, IL-6 first binds to its non-signaling membrane-bound IL-6R (also called 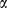-receptor subunit, IL-6R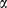) followed by recruitment of the signaling transducing receptor subunit gp130 to the complex, resulting in activation of anti-inflammatory cascades [88], [89]. Contrasting this, the IL-6 trans-signaling, pro-inflammatory pathway is activated when IL-6 binds to the soluble isoform sIL-6R and forms complexes that intercalate into the membranes of cells that contain gp130, but normally do not respond to the cytokine [48]. The trans-signaling pathway is modulated by sgp130, a circulating cleavage product of the membrane-bound receptor subunit [90]. In our model, the dual opposing actions of IL-6 were captured by assuming that the “classic” pathway activates the anti-inflammatory transcription factor STAT-3 and the trans-signaling pathway activates AP-1. IL-6 strongly induces SOCS-3 protein through STAT-3 and in turn, IL-6 signaling is selectively inhibited owing to the binding of SOCS-3 to the IL-6R subunit gp130 [91].

#### IL-10 family

IL-10 is a potent anti-inflammatory cytokine that modulates the expression of several cytokines, including IL-1, IL-6 and TNF-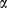 [76]. A major function of IL-10 is to control and reduce excessive immune responses during infection and autoimmunity, mainly by inhibiting the production of pro-inflammatory cytokines in macrophages and other cell types [92], [93]. IL-10 can induce the expression of SOCS-3, suggesting that the capacity of IL-10 to inhibit the expression of LPS-inducible pro-inflammatory genes may depend on SOCS-3 [91]. In this model, IL-10-induction of SOCS-3 was mediated by the transcription factor STAT-3.

#### TNF family

TNF-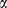 is a pro-inflammatory cytokine with important actions in immunity and inflammation, including the control of cell proliferation, differentiation, and apoptosis. Binding of TNF-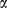 to its two receptors, TNF-RI and TNF-RII, results in recruitment of signal transducers that activate at least three distinct effectors. Through complicated signaling cascades and networks, these effectors activate caspases as well as AP-1 and NF-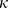
B [94], [95]. Generation of soluble TNF-RI and TNF-RII, by proteolytic cleavage, is also considered a highly regulated process. These circulating soluble receptors modify ligand actions by stabilizing TNF-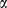 protein structure, decreasing membrane receptor number, or specifically inhibiting ligand-receptor binding [86].

### Model Calibration

Model calibration, or parameter estimation, is a key step in the development of reliable dynamic models. Given a model structure and a set of experimental data, the objective of parameter estimation is to calibrate the model to reproduce the experimental results in the best possible way. It is usually formulated as the optimization of a scalar cost function, 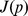, which measures the goodness of the fit with respect to the model parameters 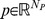. This function consists of a weighted distance measure between the experimental values corresponding to the measured variables, represented by the vector 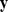, and the predicted values for those variables, represented by the vector 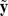. Several estimator functions have been suggested as metrics, where the weighted least-squares estimator is the most common [96]:
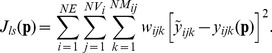
(19)


Here, 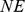 is the number of experiments, 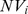 number of measured outputs in experiment 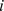, 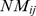 number of measurements of output 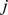 during experiment 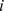, 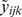 model predicted value 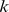 of output 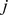 in experiment 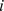, 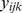 measurement 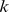 of output 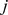 in experiment 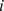, and 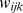 the weight of measurement 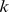 of output 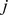 in experiment 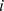.

Special attention must be paid to the selection of the weights since the optimal value of 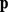 will depend on them. When a good approximation for the standard deviation of the data is available, a good choice for the weights is 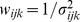 where 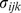 is the standard deviation of measurement 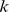 of output 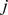 in experiment 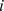. In this case, minimizing 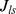 is equivalent to minimizing the Maximum Likelihood Estimator introduced by Fisher in 1912 [97], which maximizes the probability of the observed event. However, in the present study, preliminary fitting indicated that for some of the species there is no combination of parameters that accurately fits the liver and plasma data simultaneously. In order to test the hypothesis that the liver is one of the major sources of circulating cytokines, we prioritized the fit of the liver mRNA accumulation by increasing the weights of the qPCR data with respect to those of the soluble cytokines.

Due to the nonlinear nature of the model considered here, the resulting optimization problem is multimodal (non-convex). Therefore, traditional gradient based methods, like Levenberg-Marquardt or Gauss-Newton, may fail to identify the global solution and may converge to a local minimum when a better solution exists just a small distance away. Moreover, in the presence of a bad fit, there is no way of knowing if it is due to a wrong model formulation, or if it is simply a consequence of local convergence. Thus, there is a distinct need for using global optimization methods which provide more guarantee of converging to the globally optimal solution. In this work, the model was fitted to the available experimental data using SSm, a global optimization metaheuristic based on Scatter Search developed for parameter estimation in nonlinear dynamic biochemical systems [98] and available in the toolbox SensSB [22].

### Sensitivity and Correlation Analysis

A practical identifiability analysis aims to determine whether, given a model structure, the parameters of a model could be uniquely identified from the available (limited and noisy) data [99]. There are two main aspects that influence model identifiability: the sensitivity of the parameters and the correlation among them.

Sensitivity analysis indicates which parameters are the most important and therefore would have the greatest impact on the predictions of the model. To analyze how the model variables change around the best parameter set obtained, we computed local sensitivity coefficients that are the partial derivatives of the model state variables to the model parameters evaluated at the optimal point. To make these measures comparable for parameters and states of different order of magnitude, relative measures were used where the sensitivity function is normalized by the value of the parameter and the state:
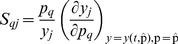
(20)


The sensitivity of all the measured states with respect to one parameter can be summarized as:
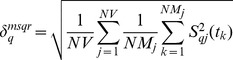
(21)


Thus, a large value of 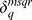 would mean that a change in the parameter 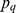 has an important effect on the model outcome. This makes the parameter identifiable with the data available if all the other parameters are fixed and the larger the sensitivity, the more accurately a single parameter can be identified. Therefore, values of critical parameters can be refined while parameters having little effect can be simplified or even ignored.

Although necessary, high parameter sensitivity is not sufficient to ensure the identifiability of the model. In the case of several parameters, the sensitivity functions of the parameters have to be linearly independent. The degree of linear dependence among the sensitivity functions can be measured by means of a correlation analysis based on the Fisher Information Matrix (FIM) as described in [100]. Correlations among parameters close to +1 or −1 mean that the parameters are not individually identifiable because a change in one parameter can be compensated by changes in the other parameters. In that case, an infinite number of parameter sets fitting the experimental data with the same accuracy would exist, thus making the confidence intervals very large. For this reason, the model should be reduced by fixing some of the parameters to their nominal values or by properly grouping some sets.

## Supporting Information

Supporting Information S1Core temperature curve for each mice and average temperature along the time axis. Different animals reach a certain temperature at different times (A) and data were collected based on matching core temperature; therefore, averaging along the time axis (B) leads to confounding results, with undistinguishable 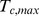 peak and differences in heating and cooling rates between the two strains.(TIF)

Supporting Information S2 Schematic diagram of the cellular network of interactions amongst HSP70, TLR4, IL-1, IL-6, IL-10, and TNF families induced by heat stroke.
(TIF)

Supporting Information S3 Model equations and SensSB files needed to reproduce the results.
(XLSX)

Supporting Information S4 Table with the list of parameters and their values.
(ZIP)

Supporting Information S5 Matlab figures with the simulated results for all the variables for WT, TNFR KO, IL-10 KO, high temperature, and LPS injection experiments.
(ZIP)

## References

[1] Sawka MN, Leon LR, Montain SJ, Sonna LA (2011) Comprehensive physiology, John Wiley & Sons, Inc., chapter Integrated physiological mechanisms of exercise performance, adaptation, and maladaptation to heat stress. pp. 1883–1928. 10.1002/cphy.c100082 23733692

[2] Borden KA, Cutter SL (2008) Spatial patterns of natural hazards mortality in the United States. International Journal of Health Geographics 7: 64–76. 19091058 10.1186/1476-072X-7-64 PMC2614968

[3] Argaud L, Ferry T, Le QH, Marfisi A, Ciorba D, et al (2007) Short- and long-term outcomes of heatstroke following the 2003 heat wave in Lyon, France. Archives of Internal Medicine 167: 2177–2183. 17698677 10.1001/archinte.167.20.ioi70147

[4] Vandentorren S, Suzan F, Medina S, Pascal M, Maulpoix A, et al (2004) Mortality in 13 French cities during the August 2003 heat wave. American Journal of Public Health 94: 1518–1520. 15333306 10.2105/ajph.94.9.1518 PMC1448485

[5] Leon LR, Helwig BG (2010) Heat stroke: Role of the systemic inflammatory response. Journal of Applied Physiology 109: 1980–1988. 20522730 10.1152/japplphysiol.00301.2010

[6] Leon L, Blaha M, DuBose D (2006) Time course of cytokine, corticosterone, and tissue injury responses in mice during heat strain recovery. Journal of Applied Physiology 100: 1400–1409. 16239608 10.1152/japplphysiol.01040.2005

[7] Bouchama A, Hammami M, Al Shail E, De Vol E (2000) Differential effects of in vitro and in vivo hyperthermia on the production of interleukin-10. Intensive Care Medicine 26: 1646–1651. 11193271 10.1007/s001340000665

[8] Bouchama A, Alsedairy S, Siddiqui S, Shail E, Rezeig M (1993) Elevated pyrogenic cytokines in heatstroke. Chest 104: 1498–1502. 8222814 10.1378/chest.104.5.1498

[9] Hammami M, Bouchama A, AlSedairy S, Shail E, AlOhaly Y, et al (1997) Concentrations of soluble tumor necrosis factor and interleukin-6 receptors in heatstroke and heatstress. Critical Care Medicine 25: 1314–1319. 9267943 10.1097/00003246-199708000-00017

[10] Hashim I, AlZeer A, AlShohaib S, AlAhwal M, Shenkin A (1997) Cytokine changes in patients with heatstroke during pilgrimage to Mecca. Mediators of Inflammation 6: 135–139. 18472847 10.1080/09629359791839 PMC2365856

[11] Bouchama A, Roberts G, Al Mohanna F, El-Sayed R, Lach B, et al (2005) Inflammatory, hemostatic, and clinical changes in a baboon experimental model for heatstroke. Journal of Applied Physiology 98: 697–705. 15475604 10.1152/japplphysiol.00461.2004

[12] Lambert G, Gisolfi C, Berg D, Moseley P, Oberley L, et al (2002) Selected contribution: Hyperthermia-induced intestinal permeability and the role of oxidative and nitrosative stress. Journal of Applied Physiology 92: 1750–1761. 11896046 10.1152/japplphysiol.00787.2001

[13] Dematte J, O’Mara K, Buescher J, Whitney C, Forsythe S, et al (1998) Near-fatal heat stroke during the 1995 heat wave in Chicago. Annals of Internal Medicine 129: 173–181. 9696724 10.7326/0003-4819-129-3-199808010-00001

[14] Helwig BG, Leon LR (2011) Tissue and circulating expression of IL-1 family members following heat stroke. Physiological Genomics 43: 1096–1104. 21828249 10.1152/physiolgenomics.00076.2011

[15] Haymaker W, Malamud N, Custer R (1947) Heat stroke - a clinic-pathologic study of 125 fatal cases. Journal of Neuropathology and Experimental Neurology 6: 209–211. 20340260

[16] Tracey K, Beutler B, Lowry S, Merryweather J, Wolpe S, et al (1986) Shock and tissue-injury induced by recombinant human cachectin. Science 234: 470–474. 3764421 10.1126/science.3764421

[17] Van Zee KJ, Kohno T, Fischer E, Rock CS, Moldawer LL, et al (1992) Tumor necrosis factor soluble receptors circulate during experimental and clinical inflammation and can protect against excessive tumor necrosis factor alpha in vitro and in vivo. Proc Natl Acad Sci U S A 89: 4845–9. 1317575 10.1073/pnas.89.11.4845 PMC49184

[18] Leon L (2006) The thermoregulatory consequences of heat stroke: are cytokines involved? Journal of Thermal Biology 31: 67–81.

[19] Vodovotz Y, Chow CC, Bartels J, Lagoa C, Prince JM, et al (2006) In silico models of acute inflammation in animals. Shock 26: 235–244. 16912648 10.1097/01.shk.0000225413.13866.fo

[20] Yang Q, Berthiaume F, Androulakis IP (2011) A quantitative model of thermal injury-induced acute inflammation. Mathematical Biosciences 229: 135–148. 20708022 10.1016/j.mbs.2010.08.003 PMC3239409

[21] Zhong J, Deaciuc J, Burikhanov R, de Villiers W (2006) Lipopolysaccharide-induced liver apoptosis is increased in interleukin-10 knockout mice. Biochimica et Biophysica Acta-Molecular Basis of Disease 1762: 468–477. 10.1016/j.bbadis.2005.12.012 16497487

[22] Rodriguez-Fernandez M, Banga J (2010) SensSB: A software toolbox for the development and sensitivity analysis of systems biology models. Bioinformatics 26: 1675–1676. 20444837 10.1093/bioinformatics/btq242

[23] Montgomery DC, Runger GC (2010) Applied statistics and probability for engineers. Wiley.

[24] Leon L, Dineen S, Blaha M, Rodriguez-Fernandez M, Clarke D (2013) Attenuated thermoregulatory, metabolic and liver acute phase protein response to heat stroke in TNF receptor knockout mice. Submitted. 10.1152/ajpregu.00127.2013 24133099

[25] Bren G, Solan N, Miyoshi H, Pennington K, Pobst L, et al (2001) Transcription of the relb gene is regulated by NF-κB. Oncogene 20: 7722–7733. 11753650 10.1038/sj.onc.1204868

[26] Sonna L, Fujita J, Gaffin S, Lilly C (2002) Invited review: Effects of heat and cold stress on mammalian gene expression. Journal of Applied Physiology 92: 1725–1742. 11896043 10.1152/japplphysiol.01143.2001

[27] Stylianou E, Saklatvala J (1998) Interleukin-1. International Journal of Biochemistry & Cell Biology 30: 1075–1079. 9785472 10.1016/s1357-2725(98)00081-8

[28] Bailly S, Fay M, Israel N, Gougerot Pocidalo M (1996) The transcription factor AP-1 binds to the human interleukin 1 alpha promoter. European Cytokine Network 7: 125–128. 8688489

[29] Weber A, Wasiliew P, Kracht M (2010) Interleukin-1 (IL-1) Pathway. Science Signaling 3: cm1. 20086235 10.1126/scisignal.3105cm1

[30] Bouchama A, Parhar R, Elyazigi A, Sheth K, Alsedairy S (1991) Endotoxemia and release of tumor-necrosis-factor and interleukin-1-alpha in acute heatstroke. Journal of Applied Physiology 70: 2640–2644. 1885459 10.1152/jappl.1991.70.6.2640

[31] Meng X, Banerjee A, Ao L, Meldrum D, Cain B, et al. (1999) Inhibition of myocardial TNF-alpha production by heat shock - A potential mechanism of stress-induced cardioprotection against postischemic dysfunction. In: Das, DK, editor, Heart in Stress. New York Acad Sciences, volume 874 of *Annals of the New York Academy of Sciences*, pp. 69–82. 10.1111/j.1749-6632.1999.tb09226.x 10415522

[32] Hasday J, Singh I (2000) Fever and the heat shock response: distinct, partially overlapping processes. Cell Stress & Chaperones 5: 471–480. 11189454 10.1379/1466-1268(2000)005<0471:fathsr>2.0.co;2 PMC312879

[33] Johnson JD, Fleshner M (2006) Releasing signals, secretory pathways, and immune function of endogenous extracellular heat shock protein 72. Journal of Leukocyte Biology 79: 425–434. 16387837 10.1189/jlb.0905523

[34] Singleton K, Wischmeyer P (2006) Oral glutamine enhances heat shock protein expression and improves survival following hyperthermia. Shock 25: 295–299. 16552363 10.1097/01.shk.0000196548.10634.02

[35] Schell M, Spitzer A, Johnson J, Lee D, Harris H (2005) Heat shock inhibits NF-κB activation in a dose- and time-dependent manner. Journal of Surgical Research 129: 90–93. 16139305 10.1016/j.jss.2005.05.025

[36] Zhang H, Xu L, Drake V, Xie L, Oberley L, et al (2003) Heat-induced liver injury in old rats is associated with exaggerated oxidative stress and altered transcription factor activation. FASEB Journal 17: 2293–5. 14500545 10.1096/fj.03-0139fje

[37] Blackwell T, Christman J (1997) The role of nuclear factor-κB in cytokine gene regulation. American Journal of Respiratory Cell and Molecular Biology 17: 3–9. 9224203 10.1165/ajrcmb.17.1.f132

[38] Cosgrove BD, Cheng C, Pritchard JR, Stolz DB, Lauffenburger DA, et al (2008) An inducible autocrine cascade regulates rat hepatocyte proliferation and apoptosis responses to tumor necrosis factor-alpha. Hepatology 48: 276–288. 18536058 10.1002/hep.22335 PMC4327877

[39] Chu B, Soncin F, Price B, Stevenson M, Calderwood S (1996) Sequential phosphorylation by mitogen-activated protein kinase and glycogen synthase kinase 3 represses transcriptional activation by heat shock factor-1. Journal of Biological Chemistry 271: 30847–30857. 8940068 10.1074/jbc.271.48.30847

[40] Zhou X, Tron V, Li G, Trotter M (1998) Heat shock transcription factor-1 regulates heat shock protein-72 expression in human keratinocytes exposed to ultraviolet B light. Journal of Investigative Dermatology 111: 194–198. 9699716 10.1046/j.1523-1747.1998.00266.x

[41] Gutenkunst RN, Waterfall JJ, Casey FP, Brown KS, Myers CR, et al (2007) Universally sloppy parameter sensitivities in systems biology models. PLoS Computational Biology 3: 1871–1878. 17922568 10.1371/journal.pcbi.0030189 PMC2000971

[42] Dehbi M, Uzzaman T, Baturcam E, Eldali A, Ventura W, et al.. (2012) Toll-like receptor 4 and high-mobility group box 1 are critical mediators of tissue injury and survival in a mouse model for heatstroke. PLOS ONE 7. 10.1371/journal.pone.0044100 PMC3433483 22962600

[43] Leon L, DuBose D, Mason C (2005) Heat stress induces a biphasic thermoregulatory response in mice. American Journal of Physiology-Regulatory Integrative and Comparative Physiology 288: R197–R204. 10.1152/ajpregu.00046.2004 15331376

[44] Gibson U, Heid C, Williams P (1996) A novel method for real time quantitative RT PCR. Genome Research 6: 995–1001. 8908519 10.1101/gr.6.10.995

[45] Livak K, Schmittgen T (2001) Analysis of relative gene expression data using real-time quantitative PCR and the 2−ΔΔCT method. Methods 25: 402–408. 11846609 10.1006/meth.2001.1262

[46] Alon U (2007) An Introduction to Systems Biology - Design Principles of Biological Circuits. Chapman & Hall/CRC.

[47] Montero-Julian F, Brailly H, Sautes C, Joyeux I, Dorval T, et al (1997) Characterization of soluble gp130 released by melanoma cell lines: A polyvalent antagonist of cytokines from the interleukin 6 family. Clinical Cancer Research 3: 1443–1451. 9815830

[48] Peake NJ, Khawaja K, Myers A, Nowell MA, Jones SA, et al (2006) Interleukin-6 signalling in juvenile idiopathic arthritis is limited by proteolytically cleaved soluble interleukin-6 receptor. Rheumatology 45: 1485–1489. 16690760 10.1093/rheumatology/kel154

[49] Levine SJ (2008) Molecular mechanisms of soluble cytokine receptor generation. Journal of Biological Chemistry 283: 14177–14181. 18385130 10.1074/jbc.R700052200 PMC2386928

[50] Lambert GP (2009) Stress-induced gastrointestinal barrier dysfunction and its inflammatory effects. Journal of Animal Science 87: E101–E108. 18791134 10.2527/jas.2008-1339

[51] Leon LR (2007) Heat stroke and cytokines. In: Neurobiology of hyperthermia, Amsterdam, Netherlands: Elsevier Science BV, volume 162 of Progress in brain research. pp. 481–524. 10.1016/S0079-6123(06)62024-4 17645934

[52] Su L, Shen L, Clayburgh DR, Nalle SC, Sullivan EA, et al (2009) Targeted epithelial tight junction dysfunction causes immune activation and contributes to development of experimental colitis. Gastroenterology 136: 551–563. 19027740 10.1053/j.gastro.2008.10.081 PMC2712351

[53] Dokladny K, Moseley P, Ma T (2006) Physiologically relevant increase in temperature causes an increase in intestinal epithelial tight junction permeability. American Journal of Physiology-Gastrointestinal and Liver Physiology 290: G204–G212. 16407590 10.1152/ajpgi.00401.2005

[54] Turner JR (2009) Intestinal mucosal barrier function in health and disease. Nature Reviews Immunology 9: 799–809. 10.1038/nri2653 19855405

[55] Yoshimura A (2006) Signal transduction of inflammatory cytokines and tumor development. Cancer Science 97: 439–447. 16734720 10.1111/j.1349-7006.2006.00197.x PMC11159428

[56] Hall D, Buettner G, Matthes R, Gisolfi C (1994) Hyperthermia stimulates nitric oxide formation: Electron paramagnetic resonance detection of NO-heme in blood. Journal of Applied Physiology 77: 548–553. 8002499 10.1152/jappl.1994.77.2.548

[57] Droge W (2002) Free radicals in the physiological control of cell function. Physiological Reviews 82: 47–95. 11773609 10.1152/physrev.00018.2001

[58] Adachi M, Liu Y, Fujii K, Calderwood SK, Nakai A, et al (2009) Oxidative stress impairs the heat stress response and delays unfolded protein recovery. PLOS ONE 4: A57–A66. 10.1371/journal.pone.0007719 PMC2777389 19936221

[59] Franca MB, Panek AD, Eleutherio ECA (2007) Oxidative stress and its effects during dehydration. Comparative Biochemistry and Physiology A-Molecular & Integrative Physiology 146: 621–631. 10.1016/j.cbpa.2006.02.030 16580854

[60] Asea A, Kraeft S, Kurt-Jones E, Stevenson M, Chen L, et al (2000) HSP70 stimulates cytokine production through a CD14-dependant pathway, demonstrating its dual role as a chaperone and cytokine. Nature Medicine 6: 435–442. 10.1038/74697 10742151

[61] Ananthan J, Goldberg A, Voellmy R (1986) Abnormal proteins serve as eukaryotic stress signals and trigger the activation of heat-shock genes. Science 232: 522–524. 3083508 10.1126/science.3083508

[62] Lepock J, Frey H, Ritchie K (1993) Protein denaturation in intact hepatocytes and isolated cellular organelles during heat-shock. Journal of Cell Biology 122: 1267–1276. 8376462 10.1083/jcb.122.6.1267 PMC2119851

[63] Peper A, Grimbergen C, Spaan J, Souren J, Van Wijk R (1998) A mathematical model of the hsp70 regulation in the cell. International Journal of Hyperthermia 14: 97–124. 9483450 10.3109/02656739809018218

[64] Baler R, Zou J, Voellmy R (1996) Evidence for a role of Hsp70 in the regulation of the heat shock response in mammalian cells. Cell Stress & Chaperones 1: 33–39. 9222587 10.1379/1466-1268(1996)001<0033:efaroh>2.3.co;2 PMC313015

[65] Santoro N, Johansson N, Thiele D (1998) Heat shock element architecture is an important determinant in the temperature and transactivation domain requirements for heat shock transcription factor. Molecular and Cellular Biology 18: 6340–6352. 9774650 10.1128/mcb.18.11.6340 PMC109220

[66] Amorim F, Moseley P (2010) Heat shock proteins and whole body physiology, Springer, chapter Heat shock protein and inflammation. pp. 57–83.

[67] Gilmore TD (2006) Introduction to NF-κB: players, pathways, perspectives. Oncogene 25: 6680–6684. 17072321 10.1038/sj.onc.1209954

[68] BarnesP, LarinM (1997) Nuclear Factor- 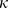 B - a pivotal transcription factor in chronic inflammatory diseases. New England Journal of Medicine 336: 1066–1071.909180410.1056/NEJM199704103361506

[69] Pahl H (1999) Activators and target genes of Rel/NF-κB transcription factors. Oncogene 18: 6853–6866. 10602461 10.1038/sj.onc.1203239

[70] Saraiva M, O’Garra A (2010) The regulation of IL-10 production by immune cells. Nature Reviews Immunology 10: 170–181. 10.1038/nri2711 20154735

[71] Covert M, Leung T, Gaston J, Baltimore D (2005) Achieving stability of lipopolysaccharideinduced NF-κB activation. Science 309: 1854–1857. 16166516 10.1126/science.1112304

[72] Cheong R, Hoffmann A, Levchenko A (2008) Understanding NF-κB signaling via mathematical modeling. Molecular Systems Biology 4: 192. 18463616 10.1038/msb.2008.30 PMC2424295

[73] Ashall L, Horton CA, Nelson DE, Paszek P, Harper CV, et al (2009) Pulsatile stimulation determines timing and specificity of NF-κB-dependent transcription. Science 324: 242–246. 19359585 10.1126/science.1164860 PMC2785900

[74] TerryAJ, ChaplainMAJ (2011) Spatio-temporal modelling of the NF- 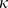 B intracellular signalling pathway: The roles of diffusion, active transport, and cell geometry. Journal of Theoretical Biology 290: 7–26.2190721210.1016/j.jtbi.2011.08.036

[75] Hess J, Angel P, Schorpp-Kistner M (2004) AP-1 subunits: quarrel and harmony among siblings. Journal of Cell Science 117: 5965–5973. 15564374 10.1242/jcs.01589

[76] Hu X, Paik P, Chen J, Yarilina A, Kockeritz L, et al (2006) IFN-gamma suppresses IL-10 production and synergizes with TLR2 by regulating GSK3 and CREB/AP-1 proteins. Immunity 24: 563–574. 16713974 10.1016/j.immuni.2006.02.014

[77] Zingarelli B, Hake P, O’Connor M, Denenberg A, Wong H, et al (2004) Differential regulation of activator protein-1 and heat shock factor-1 in myocardial ischemia and reperfusion injury: role of poly(ADP-ribose) polymerase-1. American Journal of Physiology-Heart and Circulatory Physiology 286: H1408–H1415. 14670820 10.1152/ajpheart.00953.2003

[78] Fan HY, Morgan SA, Brechun KE, Chen YY, Jaikaran ASI, et al (2011) Improving a designed photocontrolled DNA-binding protein. Biochemistry 50: 1226–1237. 21214273 10.1021/bi101432p PMC3074601

[79] Niemand C, Nimmesgern A, Haan S, Fischer P, Schaper F, et al (2003) Activation of STAT3 by IL-6 and IL-10 in primary human macrophages is differentially cytokine signaling 3. Journal of Immunology 170: 3263–3272. 10.4049/jimmunol.170.6.3263 12626585

[80] Snyder M, Huang XY, Zhang JJ (2008) Identification of novel direct Stat3 target genes for control of growth and differentiation. Journal of Biological Chemistry 283: 3791–3798. 18065416 10.1074/jbc.M706976200

[81] Levy D, Lee C (2002) What does Stat3 do? Journal of Clinical Investigation 109: 1143–1148. 11994402 10.1172/JCI15650 PMC150972

[82] Dauer D, Ferraro B, Song L, Yu B, Mora L, et al (2005) Stat3 regulates genes common to both wound healing and cancer. Oncogene 24: 3397–3408. 15735721 10.1038/sj.onc.1208469

[83] El Kasmi KC, Holst J, Coffre M, Mielke L, de Pauw A, et al (2006) General nature of the STAT3-activated anti-inflammatory response. Journal of Immunology 177: 7880–7888. 10.4049/jimmunol.177.11.7880 17114459

[84] Arend W (2002) The balance between IL-1 and IL-1Ra in disease. Cytokine & Growth Factor Reviews 13: 323–340. 12220547 10.1016/s1359-6101(02)00020-5

[85] Allan S, Rothwell N (2001) Cytokines and acute neurodegeneration. Nature Reviews Neuroscience 2: 734–744. 11584311 10.1038/35094583

[86] Heaney M, Golde D (1996) Soluble cytokine receptors. Blood 87: 847–857. 8562952

[87] Scheller J, Chalaris A, Schmidt-Arras D, Rose-John S (2011) The pro- and anti-inflammatory properties of the cytokine interleukin-6. Biochimica et Biophysica Acta (BBA) - Molecular Cell Research 1813: 878–888. 21296109 10.1016/j.bbamcr.2011.01.034

[88] Heinrich P, Behrmann I, Haan S, Hermanns H, Muller-Newen G, et al (2003) Principles of interleukin (IL)-6-type cytokine signalling and its regulation. Biochemical Journal 374: 1–20. 12773095 10.1042/BJ20030407 PMC1223585

[89] Rose-John S, Scheller J, Elson G, Jones SA (2006) Interleukin-6 biology is coordinated by membrane-bound and soluble receptors: role in inflammation and cancer. Journal of Leukocyte Biology 80: 227–236. 16707558 10.1189/jlb.1105674

[90] Barkhausen T, Tschernig T, Rosenstiel P, van Griensven M, Vonberg RP, et al (2011) Selective blockade of interleukin-6 trans-signaling improves survival in a murine polymicrobial sepsis model. Critical Care Medicine 39: 1407–1413. 21336117 10.1097/CCM.0b013e318211ff56

[91] Berlato C, Cassatella M, Kinjyo I, Gatto L, Yoshimura A, et al (2002) Involvement of suppressor of cytokine signaling-3 as a mediator of the inhibitory effects of IL-10 on lipopolysaccharide-induced macrophage activation. Journal of Immunology 168: 6404–6411. 10.4049/jimmunol.168.12.6404 12055259

[92] Otterbein L, Bach F, Alam J, Soares M, Lu H, et al (2000) Carbon monoxide has antiinflammatory effects involving the mitogen-activated protein kinase pathway. Nature Medicine 6: 422–428. 10.1038/74680 10742149

[93] Murray PJ (2007) The jak-stat signaling pathway: Input and output intergration. Journal of Immunology 178: 2623–2629. 10.4049/jimmunol.178.5.2623 17312100

[94] Cho K, Shin S, KolchW, Wolkenhauer O (2003) Experimental design in systems biology, based on parameter sensitivity analysis using a monte carlo method: A case study for the TNF*α*-mediated NF-κB signal transduction pathway. Simulation - Transactions of the Society for Modeling and Simulation International 79: 726–739.

[95] Baud V, Karin M (2001) Signal transduction by tumor necrosis factor and its relatives. Trends in Cell Biology 11: 372–377. 11514191 10.1016/s0962-8924(01)02064-5

[96] Walter E, Pronzato L (1997) Identification of parametric models from experimental data. Springer.

[97] Aldrich J (1997) R. A. Fisher and the making of maximum likelihood 1912–1922. Statistical Science 12: 162–176.

[98] Egea JA, Rodriguez-Fernandez M, Banga JR, Marti R (2007) Scatter Search for chemical and bio-process optimization. Journal of Global Optimization 37: 481–503.

[99] Jacquez JA, Greif P (1985) Numerical parameter identifiability and estimability: Integrating identifiability, estimability, and optimal sampling desing. Mathematical Biosciences 77: 201–227.

[100] Rodriguez-Fernandez M, Mendes P, Banga J (2006) A hybrid approach for efficient and robust parameter estimation in biochemical pathways. BioSystems 83: 248–265. 16236429 10.1016/j.biosystems.2005.06.016

