# Supplementary figures and images for: Modeling the Intra- and Extracellular Cytokine Signaling Pathway under Heat Stroke in the Liver
Source: PLoS One. 2013 Sep 5;8(9):e73393. doi: 10.1371/journal.pone.0073393 (PMC3764238; doi:10.1371/journal.pone.0073393)

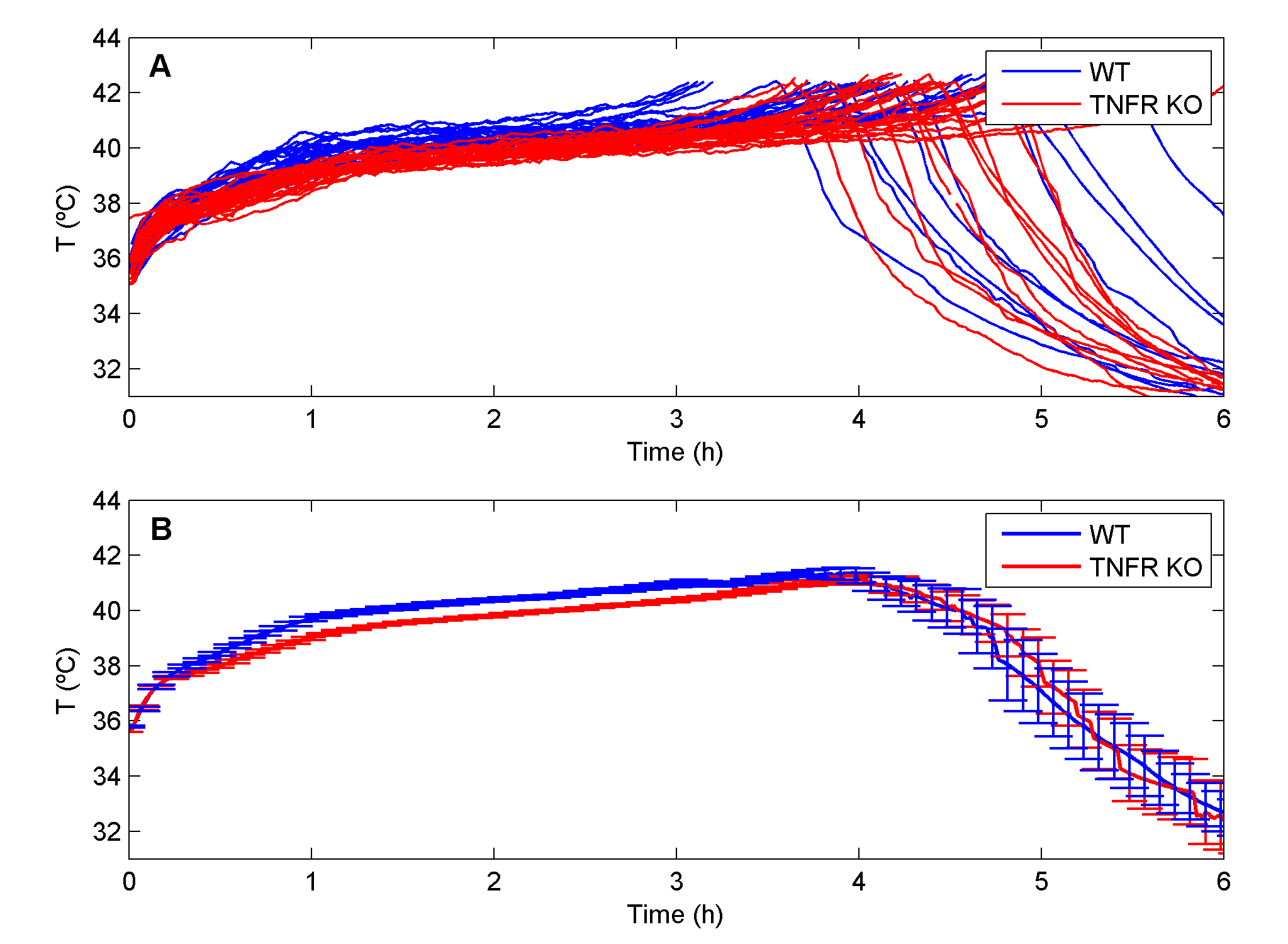

Supplement: Supporting Information S1 — Core temperature curve for each mice and average temperature along the time axis. Different animals reach a certain temperature at different times (A) and data were collected based on matching core temperature; therefore, averaging along the time axis (B) leads to confounding results, with undistinguishable peak and differences in heating and cooling rates between the two strains. (TIF) [file pone.0073393.s001.tif]

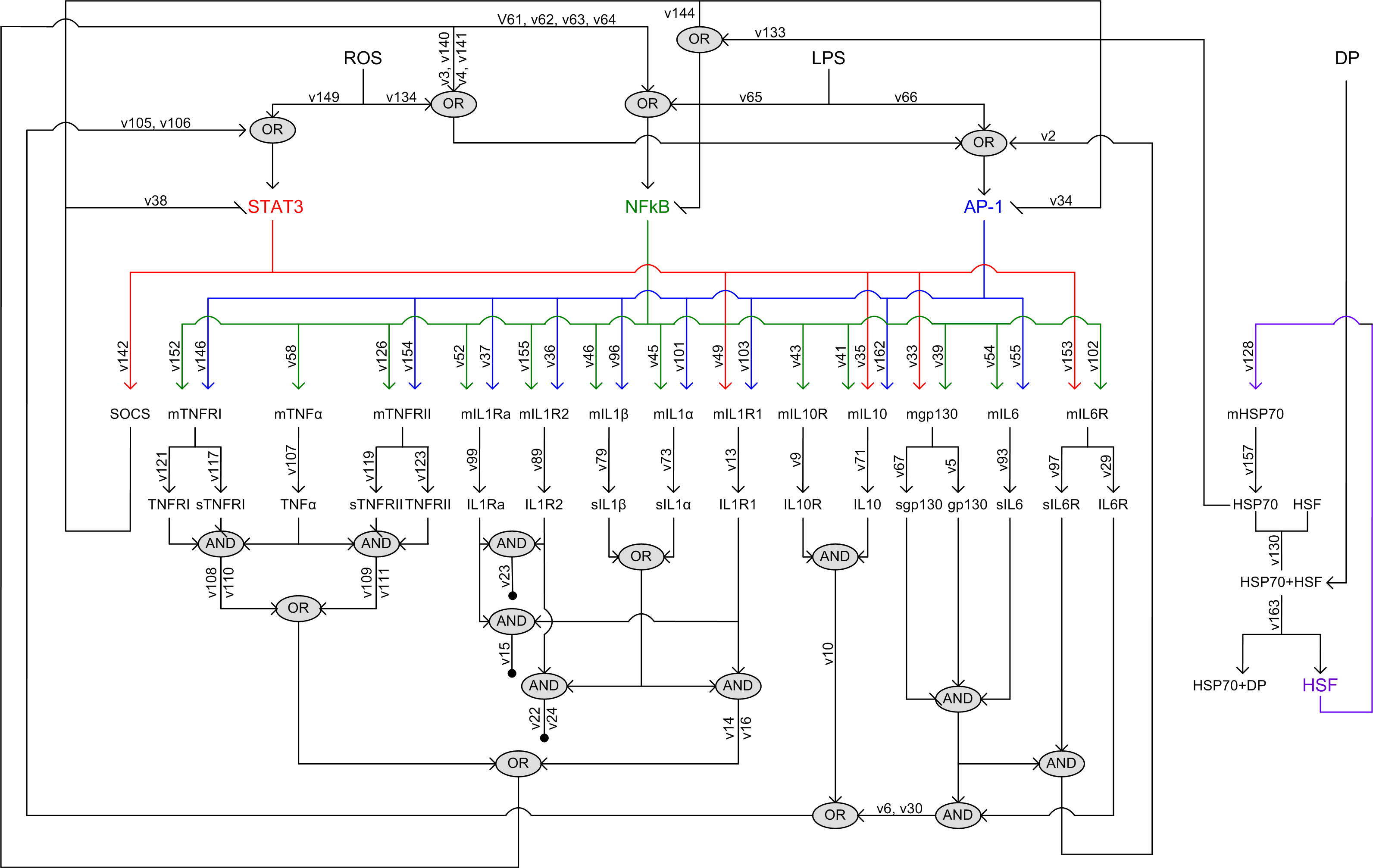

Supplement: Supporting Information S2 — Schematic diagram of the cellular network of interactions amongst HSP70, TLR4, IL-1, IL-6, IL-10, and TNF families induced by heat stroke. (TIF) [file pone.0073393.s002.tif]
